# Beta-2 Adrenergic Receptor (*ADRB2*) Gene Polymorphisms and the Risk of Asthma: A Meta-Analysis of Case-Control Studies

**DOI:** 10.1371/journal.pone.0104488

**Published:** 2014-08-11

**Authors:** Si-Qiao Liang, Xiao-Li Chen, Jing-Min Deng, Xuan Wei, Chen Gong, Zhang-Rong Chen, Zhi-Bo Wang

**Affiliations:** Department of Respiratory Medicine, First Affiliated Hospital of Guangxi Medical University, Nanning, Guangxi, China; University of British Columbia, Canada

## Abstract

**Background and Objective:**

A number of studies have assessed the relationship between beta-2 adrenergic receptor (*ADRB2*) gene polymorphisms and asthma risk. However, the results are inconsistent. A meta-analysis that focused on the association between asthma and all *ADRB2* polymorphisms with at least three case-control studies was thus performed.

**Methods:**

A literature search of the PubMed, Embase, Web of Science, CNKI, and Wangfang databases was conducted. Odds ratios with 95% confidence intervals were used to assess the strength of associations.

**Results:**

Arg16Gly, Gln27Glu, Thr164Ile, and Arg19Cys single nucleotide polymorphisms (SNPs) were identified in 46 case-control studies. The results showed that not all of the SNPs were associated with asthma in the overall population. Significant associations were found for the Arg16Gly polymorphism in the South American population via dominant model comparison (*OR* = 1.754, 95% CI = 1.179–2.609, *I^2^* = 16.9%, studies  = 2, case  = 314, control  = 237) in an analysis stratified by ethnicity. For the Gln27Glu polymorphism, a protective association was found in children via recessive model comparison (*OR* = 0.566, 95% CI = 0.417–0.769, *I^2^* = 0.0%, studies  = 11, case  = 1693, control  =  502) and homozygote genotype comparison (*OR* = 0.610, 95% CI = 0.434–0.856, *I^2^* = 0.0%, studies  = 11, case  = 1693, control  = 1502), and in adults via dominant model comparison (*OR* = 0.864, 95% CI = 0.768–0.971, *I^2^* = 46.9%, *n* = 18, case  = 3160, control  = 3433).

**Conclusions:**

None of the *ADRB2* gene polymorphisms were reproducibly associated with a risk of asthma across ethnic groups in the general population.

## Introduction

Asthma, which is characterized by variable airway obstruction caused by bronchial hyper-reactivity and airway inflammation, is one of the most common chronic respiratory diseases worldwide. The prevalence of asthma varies worldwide, ranging from 0.2% in China to 21.0% in Australia [1]. Recent studies show that asthma is a genetically related disease, with heritability estimates varying between 48% and 79% [2]. An increasing number of studies are focusing on asthma genetics research. Therefore, the identification of asthma susceptibility genes contributing to asthma pathogenesis is important. Candidate-gene linkage studies, positional cloning, and genome-wide association studies (GWAS) have already identified a large number of asthma susceptibility genes, and one of these, the beta-2 adrenergic receptor (*ADRB2*, also known as β2-AR) gene, has been extensively studied.

The β2-AR (*ADRB2*), a member of the G protein-coupled receptor (GPCR) family, is abundantly expressed on bronchial smooth muscle cells, and specifically binds and is activated by a class of ligands known as catecholamines, and epinephrine in particular [3]. The activation of β2-AR can result in the expansion of the small airways, and thus β2-AR agonists are used in first-line bronchodilator therapy in asthma [4]. The β2-AR, which can directly influence the effect of beta-2 adrenergic bronchodilator, is encoded by an intronless gene located on chromosome 5q31–32 [5]. It has been reported that *ADRB2* variants are associated with airway hypersensitivity, asthma severity, and the response to medications [6], [7]. Several single nucleotide polymorphisms (SNPs), including Arg16Gly (A46G, rs1042713), Gln27Glu (C79G, rs1042714), and Thr164Ile (C491T, rs1800888) have been identified in the coding region of the *ADRB2* gene [8]. Replacement of the base may not only alter the gene expression and function of the β2-AR, it may also alter the response to β2-AR agonist therapies and even increase the risk of asthma.

To date, various case-control studies have been conducted to investigate the relationship between *ADRB2* gene polymorphisms and asthma risk in different population groups [9]–[13], but the results have been conflicting and inconclusive. One reason for this inconsistency may be the typically small sample size of the individual studies, which may mean that there was insufficient statistical evidence to reach an agreement. A meta-analysis allows the use of all collected data to enhance the statistical power and to further prove the relationship between ADRB2 gene polymorphisms and asthma risk. To date, five meta-analyses concerning the association between ADRB2 gene polymorphisms and asthma have been reported [6], [7], [14]–[16]. However, further investigations are required for the following reasons. Three [6], [14], [15] studies were conducted in 2004 and 2005 and several additional case-control studies were performed after these were published. One study, performed in 2009, showed a relationship between *ADRB2* gene polymorphism and the response to inhaled beta-agonists in children with asthma [7]. Only one study focused on a Chinese population [16]. All of the meta-analyses described only Arg16Gly and Gln27Glu. A new meta-analysis including all ADRB2 polymorphisms that have been studied in at least three case-control studies was thus conducted to assess the overall association between ADRB2 polymorphisms and risk of asthma. This study provides a more sophisticated understanding of ADRB2 gene polymorphism and the risk of asthma.

## Materials and Methods

### Literature search

A literature search of the PubMed, Embase, Web of Science, Chinese National Knowledge Infrastructure (CNKI), and Wangfang databases (the last search was conducted on April 15, 2013) was conducted. The search strategy was as follows: “asthma” or “asthmatic” and “β2-adrenergic receptor” or “ADRB2” or “β2-AR” in combination with “polymorphism,” “mutation,” or “variant”. The searches were performed without restrictions with regard to publication date and language. Articles that were not published in English or Chinese were subsequently excluded.

### Inclusion and exclusion criteria

Studies that fulfilled the following criteria were incorporated into the meta-analysis: (1) case-control studies that evaluated the association between ADRB2 gene polymorphisms and risk of asthma; (2) the genotype distributions or allele frequency of each study was available or sufficient data could be extracted for calculating the odds ratio (*OR*) with 95% confidence interval (CI). For overlapping studies, the one with the most suitable data was selected. Studies were only excluded if they did not meet these inclusion criteria.

### Data extraction

The basic information extracted for each study was as follows: name of first author, publication year, country and ethnicity of case control, age of case, asthma definition, sample size, and genotype frequencies in cases and controls.

### Statistical analysis

Pearson's chi-square test was performed to evaluate whether the genotype distribution deviated from Hardy-Weinberg equilibrium (HWE) in the control group. Significantly deviating samples were re-assessed by 1000 time Montecarlo permutation analysis using the freely available software at http://krunch.med.yale.edu/hwsim. The *OR* with 95% CI was used to assess the strength of the association between ADRB2 polymorphism and asthma risk. The pooled *OR* for *A*DRB2 polymorphisms and asthma risk was performed for four genetic model comparisons (dominant model comparison [AA+Aa vs. aa], recessive model comparison [AA vs. Aa+aa], homozygote genotype comparison [AA vs. aa] and allele comparison [A vs. a]) to estimate the risk. In the current study, the aa genotype was a wild-type, while the AA genotype was a mutant. The Q-test and *I^2^* test were used to assess the effect of heterogeneity. Heterogeneity was considered statistically significant when Q-test (*P*<0.10) or *I^2^*>50%. If heterogeneity was indicated, data were combined according to the random-effects model; when the Q-test (*P*>0.10) or *I^2^*<50%, the fixed-effect model was used. Stratified analysis was performed by 1000 time permutation HWE P-value, ethnicity and case age to further explore HWE-specific, ethnicity-specific and age-specific effects. Sensitivity analysis was conducted by sequentially excluding one study at a time to examine the effect of each study on the combined result. Potential publication bias was investigated through the funnel plot and further assessed using Egger's test. A cumulative analysis was conducted after sorting by publication date. All statistical analyses of this meta-analysis were performed using the computer software STATA 11.0 (State Corp., College Station, TX, USA).

## Results

### Characteristics of included studies

**Figure 1:**
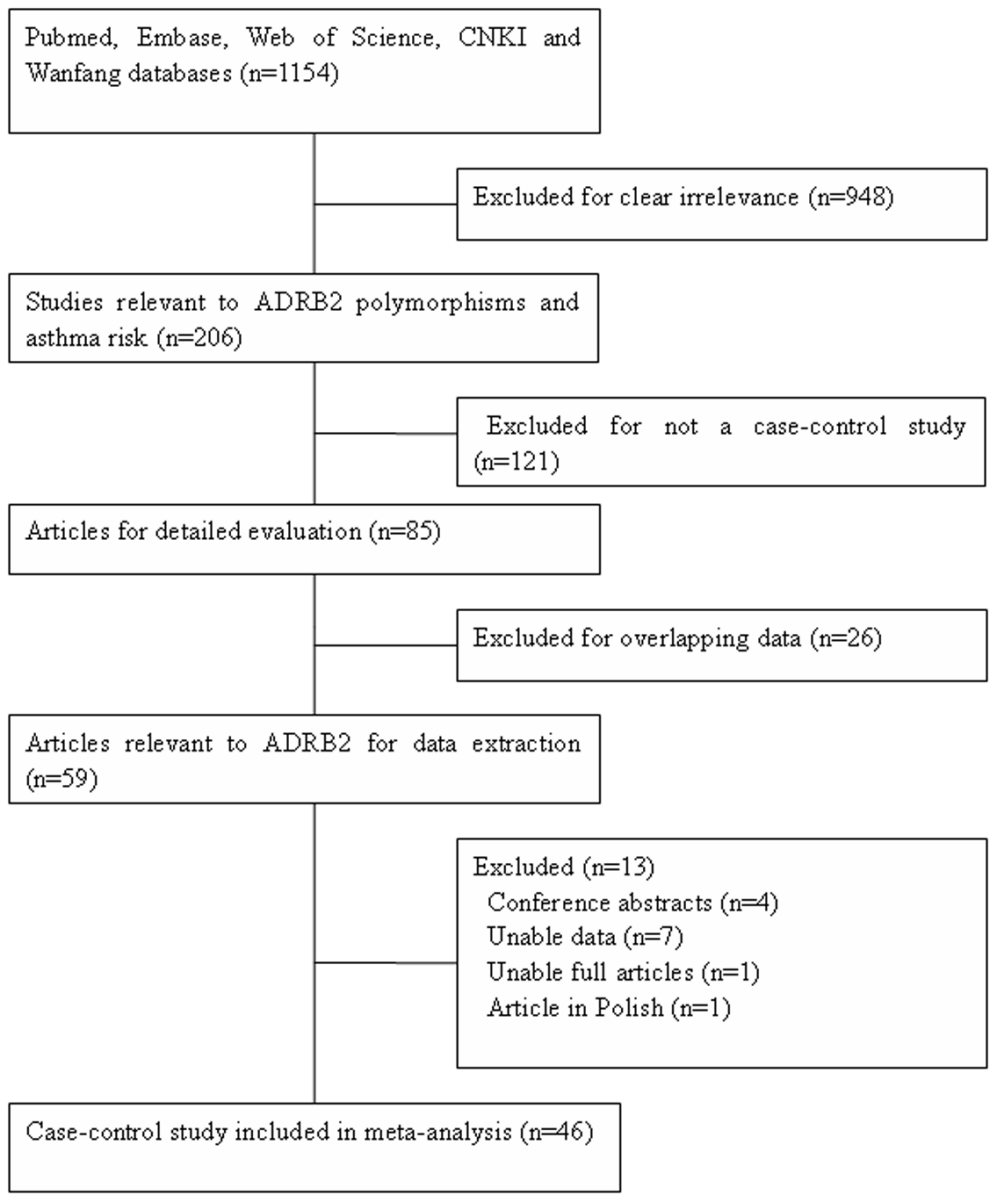
Flow diagram of included/excluded studies.

After a comprehensive search of the PubMed, Embase, Web of Science, Wanfang, and CNKI databases, 1154 articles were identified, 948 of which were subsequently excluded because they were not relevant to *ADRB2* polymorphisms and asthma risk. Thus, 206 relevant records were identified. Of these, 121 were excluded due to the lack of a case-control design. Of the remaining 85 articles, 26 were excluded due to overlapping data. Therefore, 59 articles were identified for further study. Of these 59 articles, four [17]–[20] were excluded as they were conference abstracts, seven [12], [21]–[26] did not report useable data, and one [27] was excluded because the full text was not available. In addition, one article [28] was excluded as it was in Polish. Ultimately, 46 articles [8]–[11], [13], [29]–[69] met the inclusion criteria (Figure 1). The characteristics of each article are shown in Table 1. Of these 46 articles, one [64] contained two independent studies, so the data were extracted accordingly. Furthermore, one article [65] did not provide the genotype distribution or allele frequency data, but these data were obtained from another study [15], so this article [65] was still included. Of these 46 case-control studies, three [51], [59], [64] only provided data on allele frequency and not on genotype distribution. Further analysis was performed on the *ADRB2* polymorphisms that had been reported in at least three case-control studies. A total of four SNPs met the inclusion criteria: Arg16Gly (A46G, rs1042713), Gln27Glu (C79G, rs1042714), Thr164Ile (C491T, rs1800888), and Arg19Cys (T-47C, rs1042711). Some of the included studies only focused on the Chinese population, so a meta-analysis of the Chinese population was performed independently. The genotype and allele distribution for the four SNPs are shown in Tables 2 to 5.

**Table 1:** Detailed information of each article in the meta-analysis.

First author	Year	Country	Ethnicity	Age group	Case age (year)	Control age (year)	Source of controls	Genotyping method	Cases	Control	Asthma definition
Cui LY^29^	2007	China	Asia	Adult	21–69	22–69	Population	AS-PCR/PCR-CTPP	72	60	Guidelines of prevention and treatment of bronchial asthma (Chinese Medical Association)
Ye WX^30^	2011	China	Asia	Adult	18–57	22–60	Population	AS- PCR	31	37	Guidelines of prevention and treatment of bronchial asthma (Chinese Medical Association)
Zhang XY^31^	2008	China	Asia	Children	1–17	2–13	Population	PCR-RFLP	217	50	The guidelines of treatment for bronchial asthma in children
Wang W^32^	2004	China	Asia	Adult	17–72	18–71	Hospital	SSP- PCR	123	89	Guidelines of prevention and treatment of bronchial asthma (Chinese Medical Association)
Yang Z^33^	2012	China	Asia	Children	7.7±2.6	7.69±2.55	Hospital	Sequencing	212	52	Guidelines of prevention and treatment of bronchial asthma in children(China)
Feng DX^34^	2004	China	Asia	Adult	25–63	28–63	Population	AS- PCR	74	39	Guidelines of prevention and treatment of bronchial asthma (Chinese Medical Association)
He XQ^35^	2012	China	Asia	Adult	42.5±16.2	43.39±20.70	Hospital	Sequenom MassARRAY	171	148	Guidelines of prevention and treatment of bronchial asthma (Chinese Medical Association)
Xie Y^36^	2008	China	Asia	Children	5.0±2.8	5.30±3.40	Hospital	SSP-PCR	57	62	The guidelines of treatment for bronchial asthma in children
Xing J^37^	2001	China	Asia	Adult	20–66	25–46	Population	AS- PCR	55	38	Guidelines of prevention and treatment of bronchial asthma (Chinese Medical Association)
Liu L^38^	2009	China	Asia	Adult	39.7±5.7	40.9±6.0	Population	Sequencing	120	120	Guidelines of prevention and treatment of bronchial asthma
Dai LM^39^	2002	China	Asia	Adult	42±7	46±8	Hospital	Sequencing	87	94	-
Shi XH^40^	2008	China	Asia	Both	14–66	18–56	Hospital	PCR-RFLP	48	48	Guidelines of prevention and treatment of bronchial asthma (Chinese Medical Association)
Liao W^41^	2001	China	Asia	Children	1.2–11.7	2.5–13.2	Population	PCR-RFLP	50	50	The Chinese Medical Association Respiratory Diseases Asthma Study Group
Tuerxun KLBN ^42^	2007	China	Asia	Adult	38.35±9.17	18–71	Population	SSP- PCR	76	89	Guidelines of prevention and treatment of bronchial asthma (Chinese Medical Association)
Zheng BQ^43^	2012	China	Asia	Children	0–14	0–14	Population	PCR-RFLP	198	110	Guidelines of prevention and treatment of bronchial asthma (Chinese Medical Association)
Birbian N^44^	2012	Indian	Asia	Adult	38.1±16.2	41.9±16.6	Population	PCR-RFLP	410	414	GINA (Global Initiative for Asthma) guidelines
Isaza C^45^	2012	Colombia	South America	Children	11.6±5.4	11.8±5.2	Students	Mini-sequencing	109	137	Standardised questionnaires with detailed questions on the occurrence and severity of symptoms of asthma
Kohyama K^11^	2011	Japan	Asia	Adult	49.8±15.9	47.1±13.6	Hospital	Sequence-specific thermal-elution chromatography	300	100	Global Initiative for Asthma guidelines
Fu WP^46^	2011	China	Asia	Adult	50.4±6.8	48.7±7.3	Hospital	Sequencing	238	265	Asthma was diagnosed by multiple criteria,including a history of recurrent episodes of wheezing,breathlessness,chest tightness and cough
Qiu YY^47^	2010	China	Asia	Adult	41±9	42±9	Hospital	PCR/Sequencing	201	276	Guidelines of prevention and treatment of bronchial asthma (Chinese Medical Association)
Szczepankiewicz A^48^	2009	Polish	Europe	Children	6–18	10.0±2.2	Population	PCR-RFLP	113	123	GINA recommendations,based on clinical asthma symptoms and lung function test
Llanes E^49^	2009	Spain	Europe	Adult	22.9±7.1	23–58	Population	PCR-RFLP	109	50	-
Munakata M^50^	2006	Japan	Asia	Not available	Not available	Not available	Population	PCR-RFLP	48	100	Diagnosed by symptoms and Bronchial challenge or Bronchodilator test
Tsai HJ^51^	2006		African American	Both	8–40	8–40	Hospital	Sequencing	264	176	Physician-diagnosed
Tellería JJ^52^	2005	Spain	Europe	Both	14–64	Not available	Hospital	PCR-RFLP	80	64	The American Thoracic Society guideline
Bhatnagar P^53^	2005	India	Asia	Adult	30.7±14.7	34.1±9.8	Not available	PCR	101	55	Physician-diagnosed
Gao JM^8^	2004	China	Asia	Adult	38.7±13.8	33.7±10.7	Hospital	PCR-RFLP	125	96	Guidelines of Chinese Tuberculosis and Respiratory Society
Santillan AA^54^	2003	Mexican	North America	Adult	42±14	35±12	Population	PCR-RFLP	303	604	Physician-diagnosed
Gao GK^55^	2000	China	Asia	Both	4–56	18–53	Not available	AS- PCR	58	89	Guidelines of prevention and treatment of bronchial asthma (Chinese Medical Association)
Wang Z^56^	2001	China	Asia	Adult	30.6±16.2	35.3±16.7	Population	AS- PCR	128	136	American Thoracic Society Division of Lung Disease questionnaire
Holloway JW^57^	2000	New Zealand	Oceania	Adult	31.4±1.2	32.7±1.0	Not available	PCR-RFLP	153	92	-
Reihsaus E^58^	1993	USA	Europe	Adult	23–74	Not available	Not available	PCR	51	56	Diagnosed by symptoms and medical history
Neslihan Aygun Kocabas^59^	2007	Turkish	West Asia and Southern Europe	Not available	Not available	Not available	Not available	PCR-RFLP	129	127	-
Chiang CH^9^	2012	China	Asia	Adult	46±20	44±17	Population	PCR-RFLP	476	115	The guideline of the Global Initiative for Asthma
Larocca N^60^	2012	Venezuela	South America	Adult	44.4±15.2	42.6±13.9	Not available	PCR-RFLP	105	100	GINA recommendations
Chan IH^10^	2008	China	Asia	Children	5–18	5–18	Hospital	PCR-RFLP	298	175	The American Thoracic Society guideline
Wang JY^61^	2009	China	Asia	Children	7.8±3.8	8.37±2.45	Not available	Taqman	449	512	2006 Global Initiative for Asthma guideline
Lv J^69^	2009	China	Asia	Children	3–12	18–22	Students	PCR-RFLP	192	192	2006 Global Initiative for Asthma guideline
Binaei S^62^	2003	USA	Europe	Children	Not available	Not available	Not available	PCR-RFLP	38	155	
Kotani Y^63^	1999	Japan	Asia	Adult	48.4±16.8	44.9±12.6	Not available	PCR	117	103	The American Thoracic Society criteria
Weir TD^64^	1998		Europe	Adult	34.3±13.8	41.1±17.3	Population	AS- PCR	176	146	Diagnosed by symptoms and medical history
Weir TD^64^	1998		Asia	Adult	34.3±13.8	41.1±17.3	Population	AS- PCR	176	146	Diagnosed by symptoms and medical history
Dewar JC^65^	1998	UK	Europe	Adult	18–70	18–70	Not available	AS- PCR	119	511	Physician-diagnosed
Hakonarson H^66^	2001	Iceland	Europe	Both	12–59	Not available	Hospital	PCR	324	199	European Community Respiratory Health Survey Group
Leung TF^67^	2002	China	Asia	Children	5–15	11.3±3.8	Not available	PCR	76	70	The American Thoracic Society criteria
Lin YC^68^	2003	China	Asia	Children	Not available	Not available	Students	PCR	80	69	Physician-diagnosed
Shachor J^13^	2003	Israel	Asia	Both	9–73	Not available	Not available	PCR-RFLP	66	113	The criteria of the National Heart, Lung and Blood Institute

AS-PCR: Allele-specific polymerase chain reaction, PCR-CTPP: Polymerase chain reaction with confronting two-pair primers, PCR-RFLP: polymerase chain reaction -restriction fragment length polymorphism, SSP- PCR: Sequence specific primers-polymerase chain reaction.

**Table 2:** Genotype and allele distributions in the meta-analysis for Arg16Gly (rs1042713).

First author	Year	Country	Ethnicity	Age group	Case			Control			Case		Control		HWE(*P*)	HWE(*P*)1000 permutations
					AA	AG	GG	AA	AG	GG	A	G	A	G		
Cui LY^29^	2007	China	Asia	Adult	9	55	8	12	39	9	73	71	63	57	0.019	0.038
Ye WX^30^	2011	China	Asia	Adult	5	19	7	5	26	6	29	33	36	38	0.013	0.030
Zhang XY^31^	2008	China	Asia	Children	81	111	25	19	23	8	273	161	61	39	0.814	1.000
Wang W^32^	2004	China	Asia	Adult	48	59	16	26	54	9	155	91	106	72	0.014	0.027
Yang Z^33^	2012	China	Asia	Children	78	104	30	24	23	5	260	164	71	33	0.725	1.000
Feng DX^34^	2004	China	Asia	Adult	13	35	26	6	28	5	61	87	40	38	0.006	0.016
He XQ^35^	2012	China	Asia	Adult	32	130	9	50	66	32	194	148	166	130	0.249	1.000
Xie Y^36^	2008	China	Asia	Children	14	37	6	21	34	7	65	49	76	48	0.220	0.337
Xing J^37^	2001	China	Asia	Adult	9	62	29	29	55	16	80	120	113	87	0.234	0.385
Liu L^38^	2009	China	Asia	Adult	27	59	34	23	71	26	113	127	117	123	0.044	0.082
Dai LM^39^	2002	China	Asia	Adult	33	33	21	36	33	25	99	75	105	83	0.005	0.027
Shi XH^40^	2008	China	Asia	Both	22	19	7	10	25	13	63	33	45	51	0.751	0.774
Liao W^41^	2001	China	Asia	Children	12	27	11	35	46	19	51	49	116	84	0.577	0.721
Tuerxun KLBN ^42^	2007	China	Asia	Adult	13	36	27	26	54	9	62	90	106	72	0.014	0.024
Zheng BQ^43^	2012	China	Asia	Children	77	99	28	31	55	24	253	155	117	103	0.966	1.000
Birbian N^44^	2012	Indian	Asia	Adult	62	199	149	48	188	178	323	497	284	544	0.878	0.933
Isaza C^45^	2012	Colombia	South America	Children	30	39	40	48	42	47	99	119	138	136	0.000	0.000
Kohyama K^11^	2011	Japan	Asia	Adult	40	160	100	15	50	35	240	360	80	120	0.677	0.856
Fu WP^46^	2011	China	Asia	Adult	85	88	65	106	92	67	258	218	304	226	0.000	0.000
Qiu YY^47^	2010	China	Asia	Adult	77	85	39	88	135	53	239	163	311	241	0.924	1.000
Szczepankiewicz A^48^	2009	Polish	Europe	Children	16	48	49	26	54	41	80	146	106	136	0.304	0.449
Llanes E^49^	2009	Spain	Europe	Adult	17	54	37	8	25	17	88	128	41	59	0.813	1.000
Munakata M^50^	2006	Japan	Asia	Not available	14	21	11	23	47	30	49	43	93	107	0.580	0.771
Tsai HJ^51^	2006	-	African American	Both	-	-	-	-	-	-	285	243	162	190	-	-
Tellería JJ^52^	2005	Spain	Europe	Both	13	43	24	17	29	18	69	91	63	65	0.454	0.674
Bhatnagar P^53^	2005	India	Asia	Adult	19	54	28	12	30	13	92	110	54	56	0.499	0.624
Gao JM^8^	2004	China	Asia	Adult	38	59	28	35	53	8	135	115	123	69	0.051	0.108
Santillan AA^54^	2003	Mexican	North America	Adult	56	163	84	101	318	185	275	331	520	688	0.070	0.170
Gao GK^55^	2000	China	Asia	Both	14	26	18	12	68	9	54	62	92	86	0.000	0.000
Wang Z^56^	2001	China	Asia	Adult	25	54	22	38	64	34	104	98	140	132	0.499	0.676
Holloway JW^57^	2000	New Zealand	Oceania	Adult	78	47	29	35	39	17	203	105	109	73	0.303	0.469
Reihsaus E58	1993	USA	Europe	Adult	5	19	27	7	16	33	29	73	30	82	0.042	0.174
Neslihan Aygun Kocabas^59^	2007	Turkish	West Asia and Southern Europe	Not available	-	-	-	-	-	-	91	167	108	146	-	-
Larocca N^60^	2012	Venezuela	South America	Adult	30	17	58	47	18	35	77	133	112	88	0.000	0.000
Chan IH^10^	2008	China	Asia	Children	101	135	59	51	89	33	337	253	191	155	0.597	0.700
Wang JY^61^	2009	China	Asia	Children	138	207	97	173	250	87	483	401	596	424	0.837	0.674
Lv J^69^	2009	China	Asia	Children	30	76	86	46	100	46	136	248	192	192	0.564	0.725
Binaei S^62^	2003	USA	Europe	Children	7	24	7	34	67	54	38	38	135	175	0.132	0.243
Kotani Y^63^	1999	Japan	Asia	Adult	30	52	35	28	45	30	112	122	101	105	0.201	0.342
Weir TD^64^	1998		Europe	Adult	-	-	-	-	-	-	195	125	102	66	-	-
Weir TD^64^	1998		Asia	Adult	-	-	-	-	-	-	13	19	62	62	-	-
Dewar JC^65^	1998	UK	Europe	Adult	14	50	53	74	263	180	78	156	411	623	0.158	0.251
Hakonarson H^66^	2001	Iceland	Europe	Both	45	151	127	21	85	75	241	405	127	235	0.677	0.874
Leung TF^67^	2002	China	Asia	Children	25	38	13	22	37	11	88	64	81	59	0.483	0.675
Lin YC^68^	2003	China	Asia	Children	34	35	11	27	25	17	103	57	79	59	0.031	0.104
Shachor J^13^	2003	Israel	Asia	Both	11	38	17	26	52	35	60	72	104	122	0.433	0.531

**Table 3:** Genotype and allele distributions in the meta-analysis for Gln27Glu (rs1042714).

First author	Year	Country	Ethnicity	Age group	Case			Control			Case		Control		HWE(*P*)	HWE(*P*) 1000 permutations
					CC	CG	GG	CC	CG	GG	C	G	C	G		
Cui LY^29^	2007	China	Asia	Adult	52	11	9	52	4	4	115	29	108	12	0.000	0.024
Ye WX^30^	2011	China	Asia	Adult	10	17	4	14	19	4	37	25	47	27	0.511	0.763
Zhang XY^31^	2008	China	Asia	Children	54	119	44	8	24	18	227	207	40	60	1.000	1.000
Wang W^32^	2004	China	Asia	Adult	73	33	17	52	27	10	179	67	131	47	0.038	0.153
Yang Z^33^	2012	China	Asia	Children	183	28	1	52	0	0	394	30	104	0	-	-
Feng DX^34^	2004	China	Asia	Adult	25	39	10	15	20	4	89	59	50	28	0.475	0.510
Xie Y^36^	2008	China	Asia	Children	49	5	3	51	4	7	103	11	106	18	0.000	0.000
Xing J^37^	2001	China	Asia	Adult	35	58	7	23	74	3	128	72	120	80	0.000	0.000
Dai LM^39^	2002	China	Asia	Adult	71	13	3	76	14	4	155	19	166	22	0.007	0.015
Liao W^41^	2001	China	Asia	Children	26	20	4	52	36	12	72	28	140	60	0.153	0.327
Tuerxun KLBN ^42^	2007	China	Asia	Adult	44	29	3	52	34	3	117	35	138	40	0.363	0.646
Birbian N^44^	2012	Indian	Asia	Adult	224	146	40	203	168	43	594	226	574	254	0.350	0.465
Isaza C^45^	2012	Colombia	South America	Children	76	29	4	103	29	5	181	37	235	39	0.120	0.322
Fu WP^46^	2011	China	Asia	Adult	179	38	21	209	37	19	396	80	455	75	0.000	0.001
Qiu YY^47^	2010	China	Asia	Adult	166	32	3	226	45	5	364	38	497	55	0.129	0.386
Szczepankiewicz A^48^	2009	Polish	Europe	Children	31	58	24	39	48	36	120	106	126	120	0.015	0.540
Llanes E^49^	2009	Spain	Europe	Adult	49	40	18	24	22	4	138	76	70	30	0.736	0.783
Munakata M^50^	2006	Japan	Asia	Not available	39	6	1	86	14	0	84	8	186	14	0.452	1.000
Tsai HJ^51^	2005	Spain	Europe	Both	27	39	14	30	20	14	93	67	80	48	0.008	0.420
Gao JM^8^	2004	China	Asia	Adult	46	76	3	39	56	1	168	82	134	58	0.000	0.002
Santillan AA^54^	2003	Mexican	North America	Adult	241	53	9	385	202	17	535	71	972	236	0.117	0.248
Gao GK^55^	2000	China	Asia	Both	20	32	6	32	49	8	72	44	113	65	0.077	0.171
Wang Z^56^	2001	China	Asia	Adult	108	19	1	113	22	1	235	21	248	24	0.950	0.303
Holloway JW^57^	2000	New Zealand	Oceania	Adult	28	76	49	19	37	35	132	174	75	107	0.125	0.235
Reihsaus E^58^	1993	USA	Europe	Adult	13	26	12	17	23	16	52	50	57	55	0.182	0.384
Chiang CH^9^	2012	China	Asia	Adult	400	66	10	85	26	1	866	86	196	28	0.517	0.743
Larocca N^60^	2012	Venezuela	South America	Adult	37	57	11	30	60	10	131	79	120	80	0.012	0.060
Chan IH^10^	2008	China	Asia	Children	232	43	19	133	19	21	507	81	285	61	0.000	0.000
Wang JY^61^	2009	China	Asia	Children	359	84	5	425	77	9	802	94	927	95	0.016	0.201
Binaei S^62^	2003	USA	Europe	Children	23	12	2	107	36	12	58	16	250	60	0.001	0.039
Kotani Y^63^	1999	Japan	Asia	Adult	94	23	0	89	14	0	211	23	192	14	0.459	1.000
Weir TD^64^	1998	-	Europe	Adult	-	-	-	-	-	-	174	136	101	67	-	-
Weir TD^64^	1998	-	Asia	Adult	-	-	-	-	-	-	26	6	91	33	-	-
Dewar JC^65^	1998	UK	Europe	Adult	33	51	35	134	271	106	117	121	539	483	0.149	0.225
Hakonarson H^66^	2001	Iceland	Europe	Both	92	173	59	48	112	39	357	291	208	190	0.071	0.149
Leung TF^67^	2002	China	Asia	Children	64	12	0	55	15	0	140	12	125	15	0.315	0.642
Lin YC^68^	2003	China	Asia	Children	65	15	0	54	14	1	145	15	122	16	0.932	1.000
Shachor J^13^	2003	Israel	Asia	Both	33	27	4	53	49	9	93	35	155	67	0.617	0.671

**Table 4:** Genotype and allele distributions in the meta-analysis for Thr164Ile (rs1800888).

First author	Year	Country	Ethnicity	Age group	Case			Control			Case		Control		HWE(*P*)	HWE(*P*)1000 permutations
					CC	CT	TT	CC	CT	TT	C	T	C	T		
Yang Z^33^	2012	China	Asia	Children	211	1	0	52	0	0	423	1	104	0	-	-
Gao JM^8^	2004	China	Asia	Adult	56	67	2	48	48	0	179	71	144	48	0.001	0.021
Gao GK^55^	2000	China	Asia	Both	6	48	4	27	47	15	60	56	101	77	0.475	0.546
Reihsaus E^58^	1993	USA	Europe	Adult	51	0	0	53	3	0	102	0	109	3	0.837	1.000

**Table 5:** Genotype and allele distributions in the meta-analysis for Arg19Cys (rs1042711).

First author	Year	Country	Ethnicity	Age group	Case			Control			Case		Control		HWE(*P*)	HWE(*P*) 1000 permutations
					TT	CT	CC	TT	CT	CC	T	C	T	C		
Fu WP^46^	2011	China	Asia	Adult	162	69	7	199	61	5	393	83	459	71	0.897	1.000
Qiu YY^47^	2010	China	Asia	Adult	166	32	3	226	45	5	364	38	497	55	0.129	0.384
Szczepankiewicz A^48^	2009	Polish	Europe	Children	51	41	21	57	49	17	143	83	163	83	0.227	0.407
Tsai HJ^51^	2006	-	African American	Both	-	-	-	-	-	-	454	74	289	63	-	-

### HWE for included studies

The HWE for each included study was calculated by chi-square test. The P-value of the genotype distribution in each control group is shown in Tables 2 to 5. As some of the included studies were not in HWE, a stratified analysis according to the P-value for the Arg16Gly and Gln27Glu polymorphisms was conducted. The results are shown in Table 6.

**Table 6:** Main results of pooled ORs in the meta-analysis.

SNP	Groups	Dominant model comparison			Recessive model comparison			Homozygote genotype comparison			Allelic comparison		
		OR (95%CI)	*P*_(Z)_	*I^2^*	OR (95%CI)	*P*_(Z)_	*I^2^*	OR (95%CI)	*P*_(Z)_	*I^2^*	OR (95%CI)	*P*_(Z)_	*I^2^*
Arg16Gly	Total	1.069 (0.978–1.167)	0.142	46.4%	1.111(0.949–1.300)	0.192	64.2%	1.155(0.969–1.376)	0.108	54.3%	1.074( 0.987–1.168)	0.098	58.5%
(rs1042713)	Adult	1.077 (0.956–1.213)	0.225	51.8%	1.170(0.942–1.454)	0.155	67.9%	1.230(0.965–1.569)	0.094	57.9%	1.110 (0.992–1.242)	0.069	57.3%
	Children	1.122 (0.970–1.299)	0.121	21.5%	1.061(0.798–1.410)	0.685	61.4%	1.158(0.851–1.575)	0.350	53.9%	1.092(0.930–1.282)	0.282	60.0%
	Both	0.846(0.607–1.1815)	0.326	66.7%	1.064(0.617–1.833)	0.824	67.9%	0.946(0.526–1.702)	0.853	51.4%	0.896(0.704–1.140)	0.372	56.7%
	Not available	0.683 (0.312–1.492)	0.339	-	0.733(0.329–1.634)	0.448	-	0.602(0.231–1.571)	0.300	-	1.045(0.595–1.834)	0.878	70.9%
	Asia	1.055(0.954–1.168)	0.297	49.2%	1.122(0.913–1.380)	0.275	68.6%	1.139(0.914–1.420)	0.247	58.8%	1.074(0.970–1.189)	0.167	57.1%
	Europe	1.205(0.910–1.596)	0.192	0.0%	1.055(0.793–1.404)	0.713	41.6%	1.202(0.881–1.640)	0.245	1.1%	1.079(0.929–1.252)	0.319	64.6%
	South America	1.754(1.179–2.609)	0.006	16.9%	1.583(0.778–3.221)	0.205	70.6%	1.880(0.999–3.539)	0.050	51.8%	1.627(0.913–2.897)	0.098	78.7%
	North America	0.886 (0.618–1.270)	0.509	-	0.869(0.640–1.179)	0.366	-	0.819(0.540–1.241)		-	0.910(0.748–1.107)		-
	Oceania	0.609(0.359–1.032)	0.065	-	1.010(0.520–1.962)	0.977	-	0.765(0.373–1.572)	0.466	-	0.772(0.529–1.128)	0.181	-
	China	1.093(0.914–1.305)	0.330	55.4%	1.199(0.929–1.548)	0.162	71.2%	1.209(0.929–1.573)	0.159	62.6%	1.104(0.980–1.245)	0.105	60.6%
	HWE (P>0.05)	1.041(0.943–1.149)	0.339	47.0%	1.003(0.850–1.183)	0.973	60.7%	1.058(0.869–1.287)	0.576	54.4%	1.041(0.942–1.152)	0.428	58.9%
	HWE (P<0.05)	1.186(0.972–1.446)	0.196	46.0%	1.673(1.136–2.466)	0.009	64.7%	1.578(1.122–2.221)	0.009	38.0%	1.185(0.997–1.409)	0.054	53.2%
Gln27Glu	Total	0.925(0.843–1.014)	0.097	34.8%	0.935(0.805–1.086)	0.380	0.0%	0.936(0.793–1.105)	0.435	0.0%	0.947(0.883–1.015)	0.122	25.9%
(rs1042714)	Adult	0.864(0.768–0.971)	0.014	46.9%	1.158(0.952–1.408)	0.143	0.0%	1.123(0.905–1.392)	0.292	0.0%	0.955(0.875–1.042)	0.302	37.9%
	Children	1.061(0.885–1.274)	0.521	3.0%	0.566(0.417–0.769)	0.000	0.0%	0.610(0.434–0.856)	0.004	0.0%	0.912(0.788–1.056)	0.218	28.4%
	Both	0.969(0.734–1.278)	0.822	23.3%	0.890(0.624–1.271)	0.522	0.0%	0.878(0.58–1.318)	0.531	-	0.955(0.793–1.150)	0.624	0.0%
	Not available	1.103(0.413–2.947)	0.846	-	6.626(0.265–165.798)	0.250	-	6.570(0.262–164.864)	0.252	-	1.265(0.511–3.131)	0.611	-
	Asia	0.957(0.854–1.073)	0.451	7.0%	0.886(0.713–1.101)	0.275	0.0%	0.884(0.704–1.110)	0.289	0.0%	0.949(0.866–1.040)	0.262	12.1%
	Europe	1.057(0.853–1.309)	0.614	0.0%	1.023(0.801–1.307)	0.853	35.9%	1.032(0.775–1.373)	0.829	0.0%	1.047(0.918–1.195)	0.493	0.0%
	South America	1.028(0.685–1.543)	0.893	34.6%	1.038(0.491–2.196)	0.922	0.0%	0.954(0.431–2.111)	0.908	0.0%	1.023(0.751–1.392)	0.887	0.0%
	North America	0.452(0.327–0.626)	0.000	-	1.057(0.466–2.400)	0.895	-	0.846(0.371–1.928)	0.690	-	0.547(0.411–0.727)	0.000	-
	Oceania	1.178(0.615–2.258)	0.622	-	0.754(0.438–1.296)	0.307	-	0.950(0.460–1.964)	0.890	-	0.924(0.637–1.340)	0.677	-
	China	0.984(0.863–1.122)	0.813	9.2%	0.867(0.674–1.117)	0.270	0.0%	0.894(0.684–1.168)	0.411	0.0%	0.967(0.870–1.075)	0.536	18.9%
	HWE (P>0.05)	0.895(0.807–0.992)	0.035	32.0%	0.940(.798–1.108)	0.463	0.0%	0.941(0.781–1.133)	0.520	0.0%	0.925(0.855–1.001)	0.053	18.5%
	HWE (P<0.05)	1.042(0.844–1.287)	0.704	28.3%	0.913(0.633–1.315)	0.624	26.9%	0.919(0.635–1.329)	0.652	15.5%	1.006(0.853–1.186)	0.944	38.4%
Thr164Ile	Total	1.460(0.544–3.916)	0.452	54.3%	0.772(0.089–6.684)	0.814	50.7%	1.502(0.416–5.419)	0.535	0.0%	1.173(0.858–1.603)	0.318	0.0%
(rs1800888)													
Arg19Cys	Total	1.165(0.898–1.510)	0.250	0.0%	1.344(0.773–2.335)	0.295	0.0%	1.340(0.754–2.381)	0.318	0.0%	1.039(0.860–1.254)	0.691	49.4%
(rs1042711)													

### Meta-analysis of *ADRB2* polymorphisms and asthma

#### Meta-analysis of Arg16Gly variants and asthma

**Figure 2:**
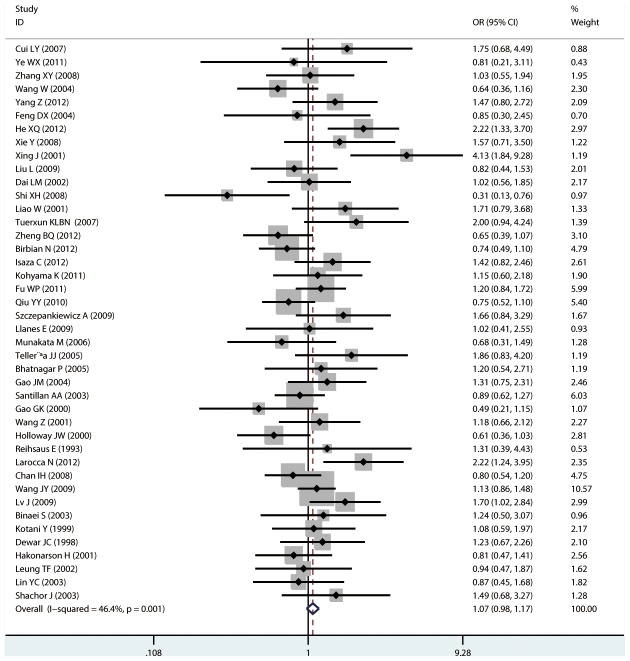
Forest plots of the association between the Arg16Gly (rs1042713) polymorphism and risk of asthma in dominant model comparison.

**Figure 3:**
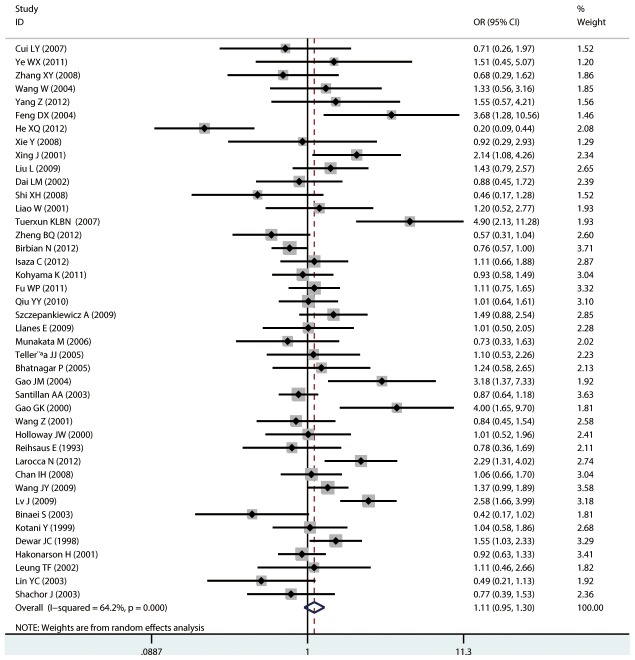
Forest plots of the association between the Arg16Gly (rs1042713) polymorphism and risk of asthma in recessive model comparison.

**Figure 4:**
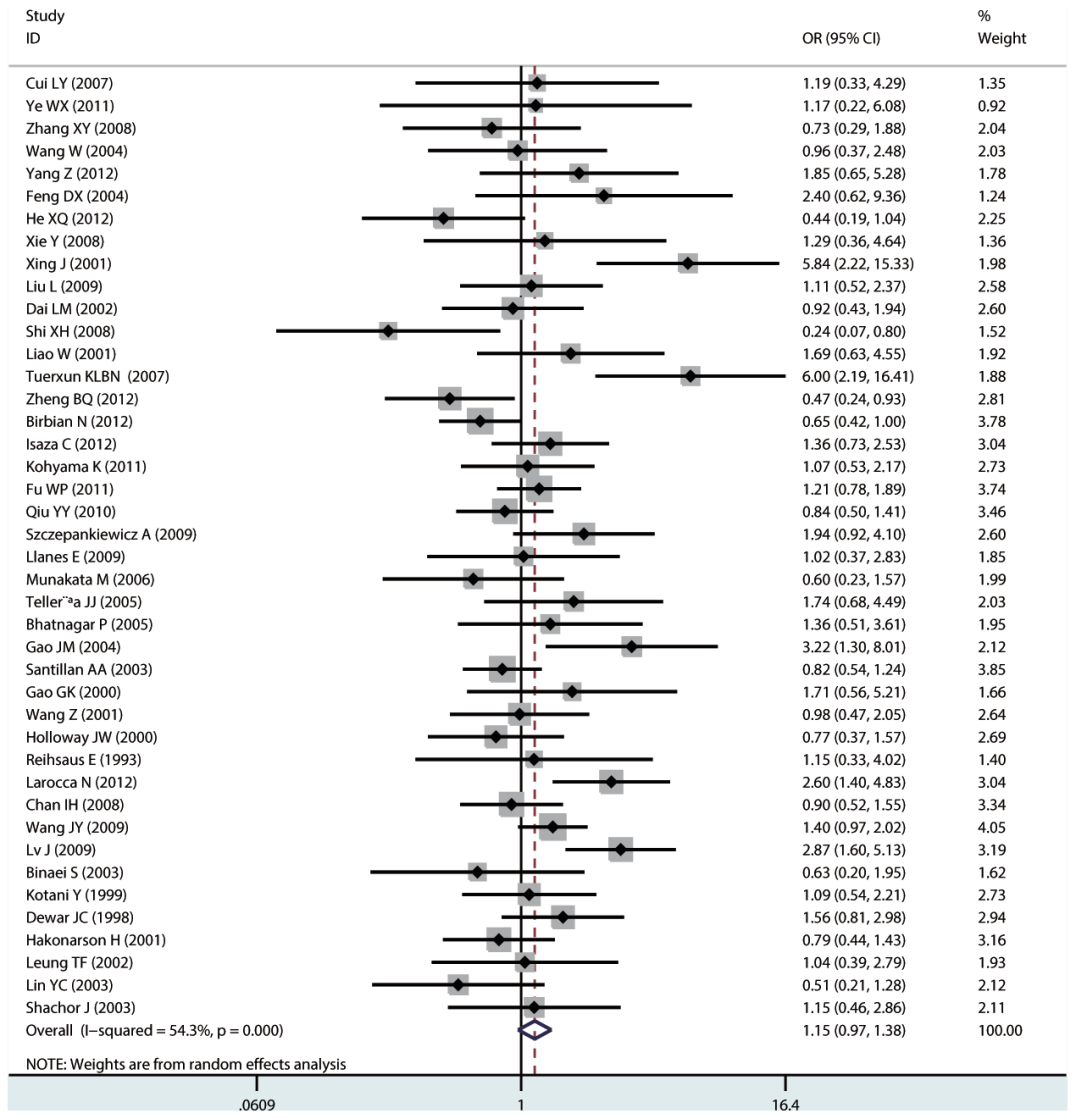
Forest plots of the association between the Arg16Gly (rs1042713) polymorphism and risk of asthma in homozygote genotype comparison.

**Figure 5:**
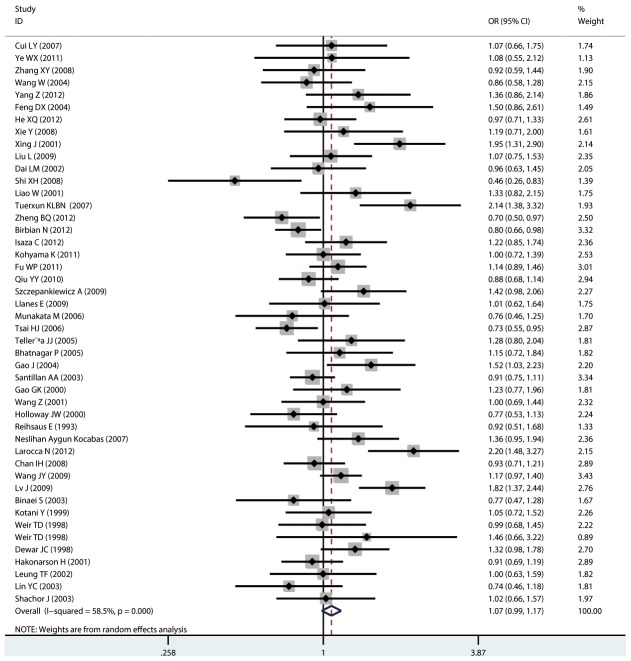
Forest plots of the association between the Arg16Gly (rs1042713) polymorphism and risk of asthma in allele comparison.

For Arg16Gly, there was no significant association in any of the genetic model comparisons in the overall population (Figures 2 to 5). In the analysis stratified by ethnicity, a significant association was found in the South American population in the dominant model comparison (*OR* = 1.754, 95% CI = 1.179–2.609, *I^2^* = 16.9%, studies  = 2, case  = 314, control  = 237), but not in the other genetic comparisons or other ethnic groups. In the Chinese population, there was no significant association in any of the genetic model comparisons. The results are shown in Table 6.

#### Meta-analysis of Gln27Glu variants and asthma

**Figure 6:**
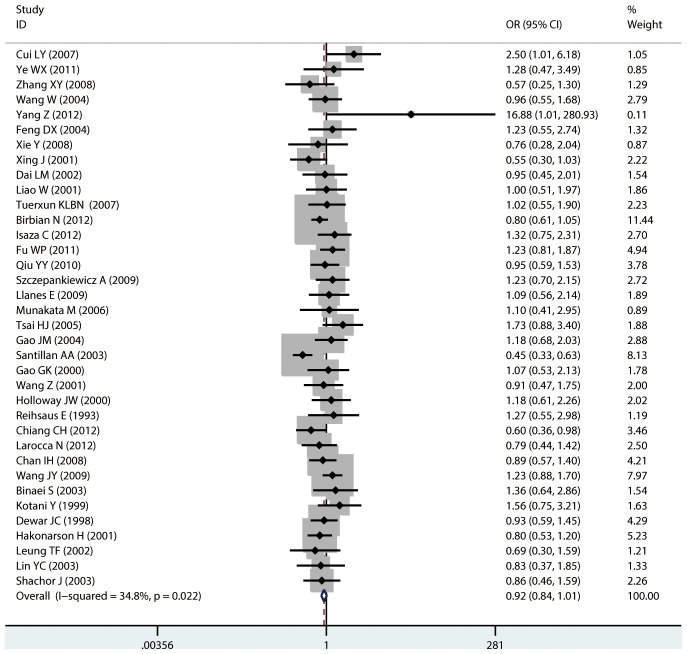
Forest plots of the association between the Gln27Glu (rs1042714) polymorphism and risk of asthma in dominant model comparison.

**Figure 7:**
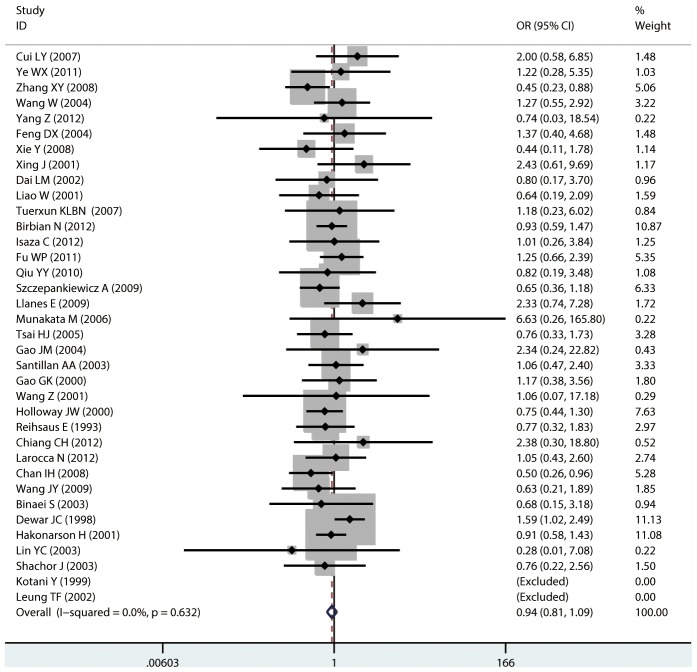
Forest plots of the association between the Gln27Glu (rs1042714) polymorphism and risk of asthma in recessive model comparison.

**Figure 8:**
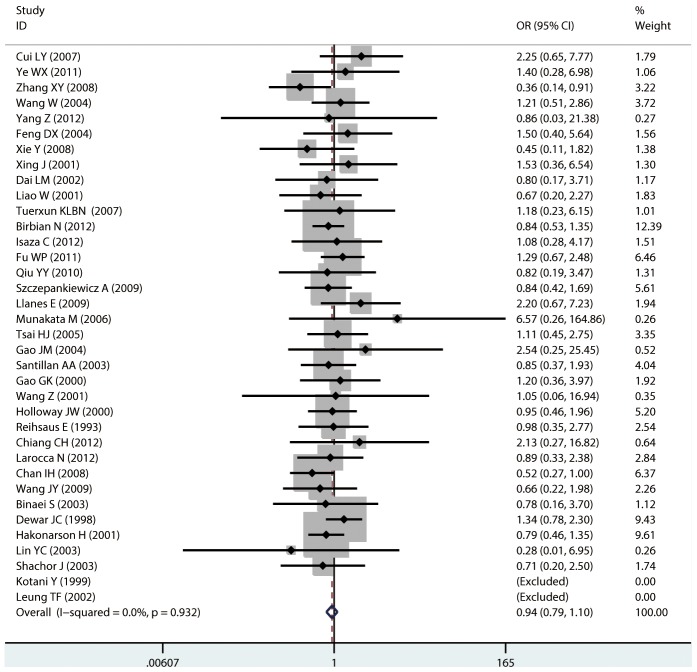
Forest plots of the association between the Gln27Glu (rs1042714) polymorphism and risk of asthma in homozygote genotype comparison.

**Figure 9:**
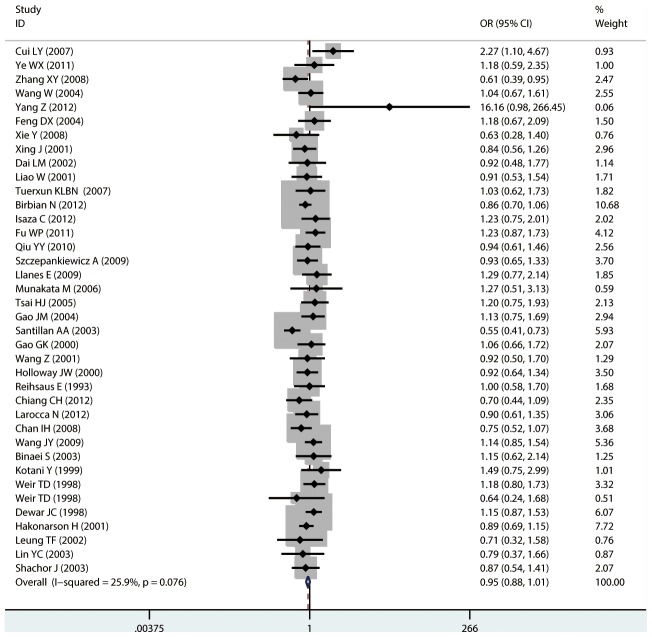
Forest plots of the association between the Gln27Glu (rs1042714) polymorphism and risk of asthma in allele comparison.

For Gln27Glu, no evidence of an association with asthma risk was found in the overall population in any of the genetic model comparisons (Figures 6 to 9). In the analysis stratified by case age, a protective association was found in children only in the recessive model comparison (*OR* = 0.566, 95% CI = 0.417–0.769, *I ^2^* = 0.0%, studies  = 11, case  = 1693, control  = 1502) and homozygote genotype comparison (*OR* = 0.610, 95% CI = 0.434–0.856, *I^2^* = 0.0%, studies  = 11, case  = 1693, control  = 1502), and in adults only in the dominant model comparison (*OR* = 0.864, 95% CI = 0.768–0.971, *I^2^* = 46.9% *n* = 18, case  = 3160, control  = 3433). In the Chinese population, there was no significant association in any of the genetic model comparisons. The results are shown in Table 6.

#### Meta-analysis of Thr164Ile variants and asthma

For Thr164Ile, only four case-control studies were included, so no stratified analysis was performed. There was no evidence of an association with asthma risk in any of the genetic models in the overall population. The results are shown in Table 6.

#### Meta-analysis of Arg19Cys variants and asthma

For Arg19Cys, only three case-control studies provided genotype distribution data, therefore no stratified analysis was conducted. No significant association was found in the overall population in any of the genetic models. The results are shown in Table 6.

### Cumulative meta-analysis

**Figure 10:**
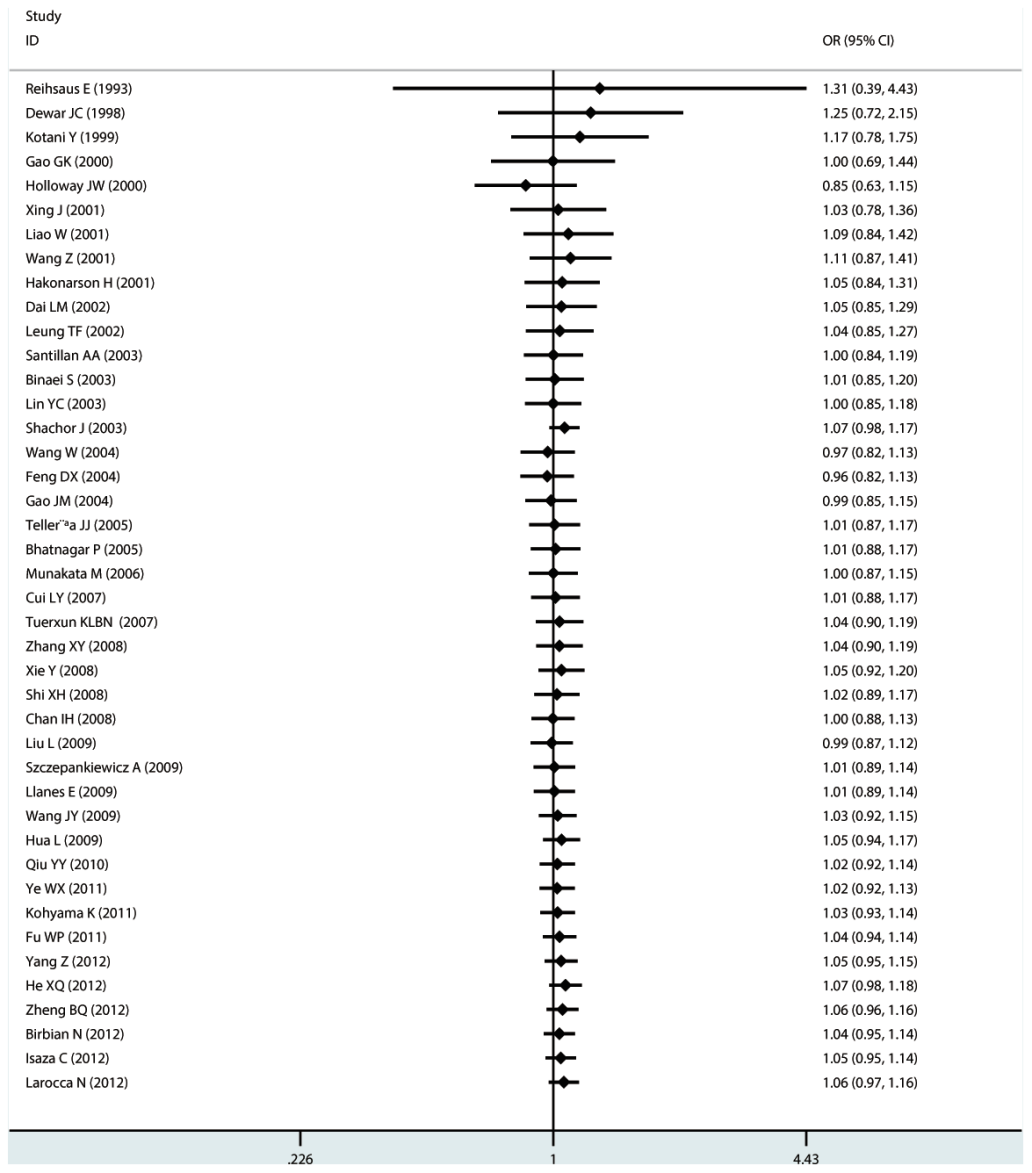
Forest plots of cumulative meta-analysis of Arg16Gly (rs1042713) in association with asthma by published year under dominant model comparison.

**Figure 11:**
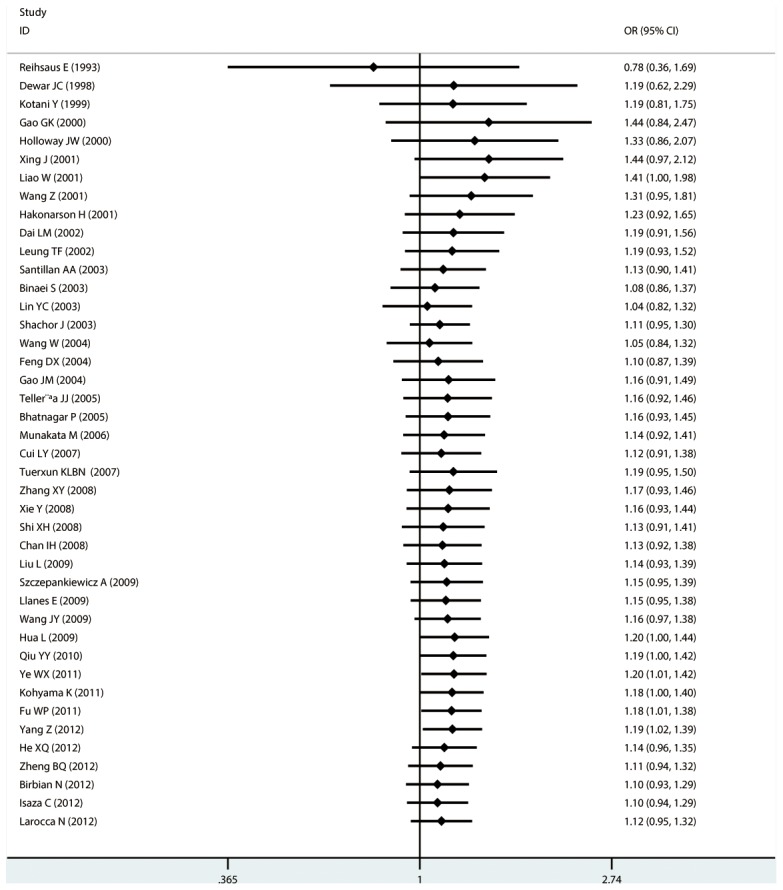
Forest plots of cumulative meta-analysis of Arg16Gly (rs1042713) in association with asthma by published year under recessive model comparison.

**Figure 12:**
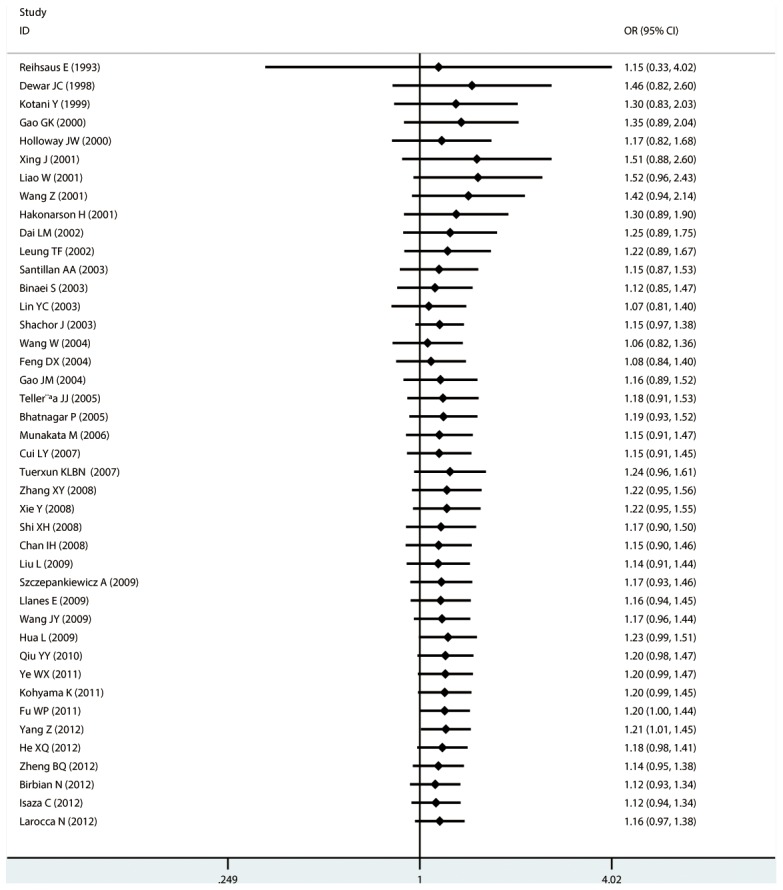
Forest plots of cumulative meta-analysis of Arg16Gly (rs1042713) in association with asthma by published year under homozygote genotype comparison.

**Figure 13:**
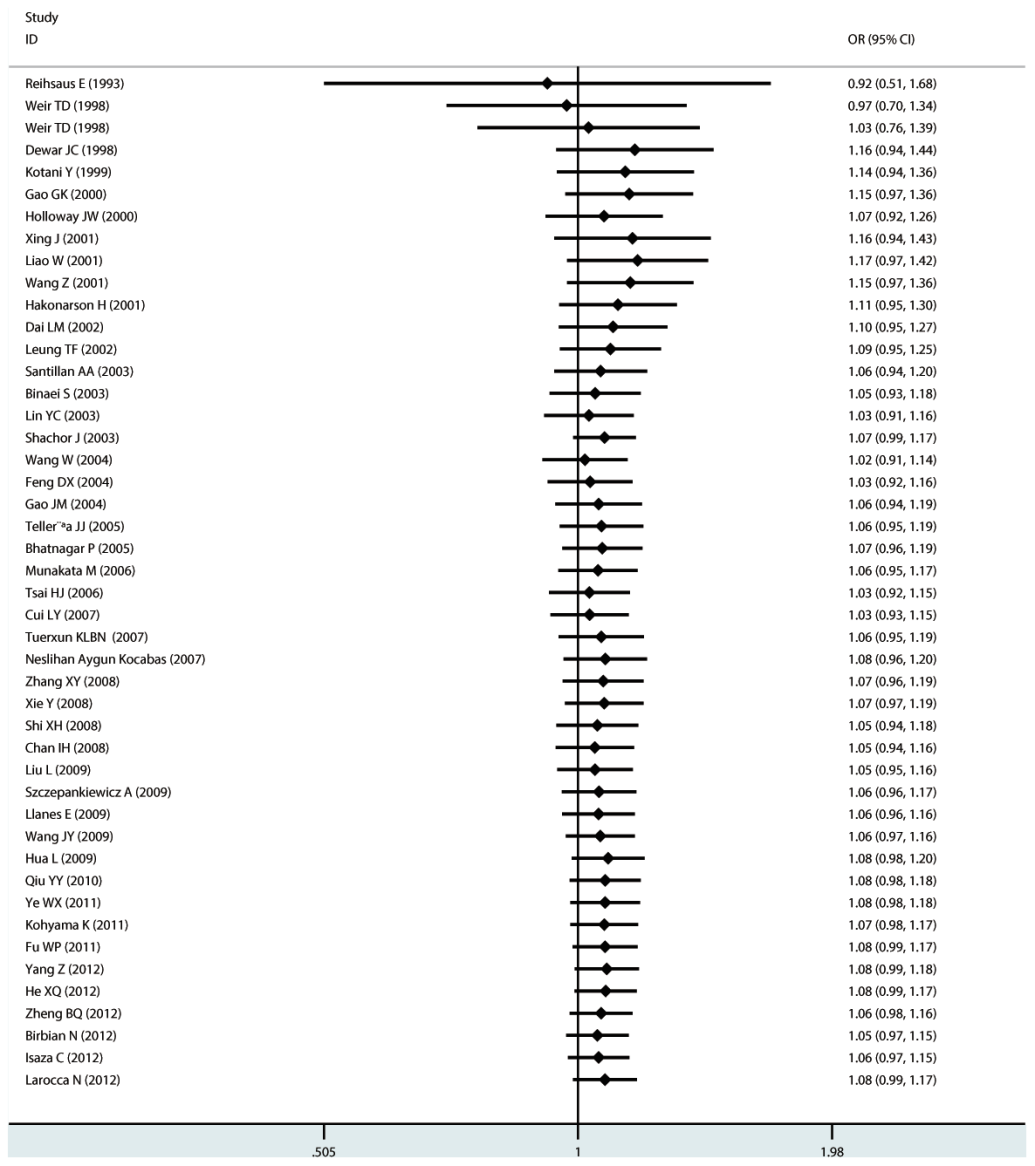
Forest plots of cumulative meta-analysis of Arg16Gly (rs1042713) in association with asthma by published year under allele comparison.

**Figure 14:**
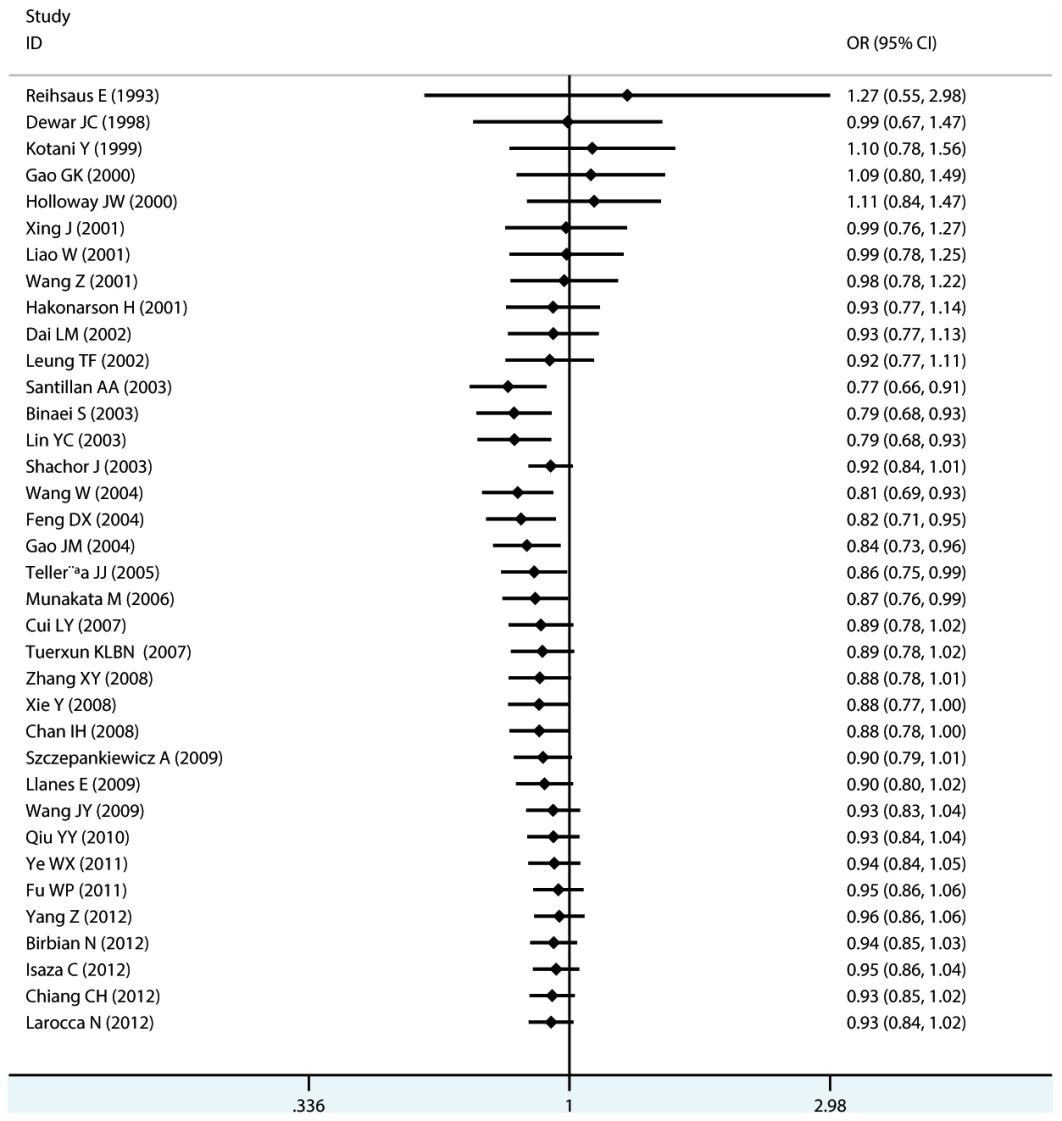
Forest plots of cumulative meta-analysis of Gln27Glu (rs1042714) in association with asthma by published year dominant model comparison.

**Figure 15:**
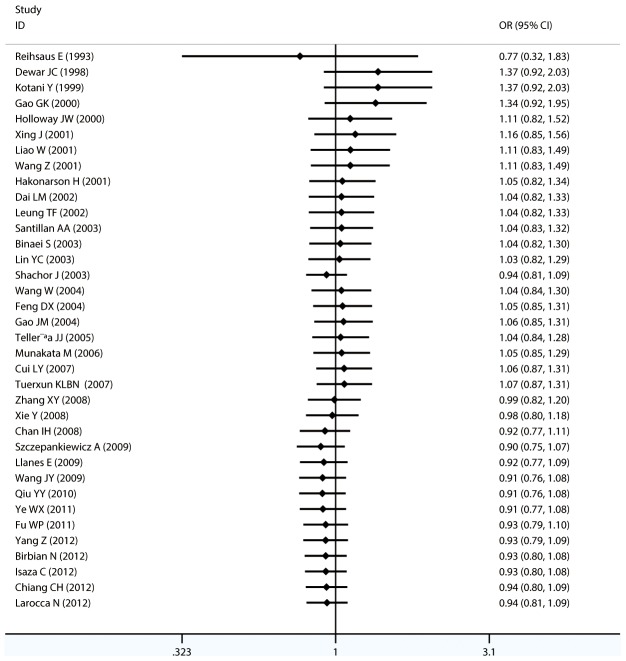
Forest plots of cumulative meta-analysis of Gln27Glu (rs1042714) in association with asthma by published year under recessive model comparison.

**Figure 16:**
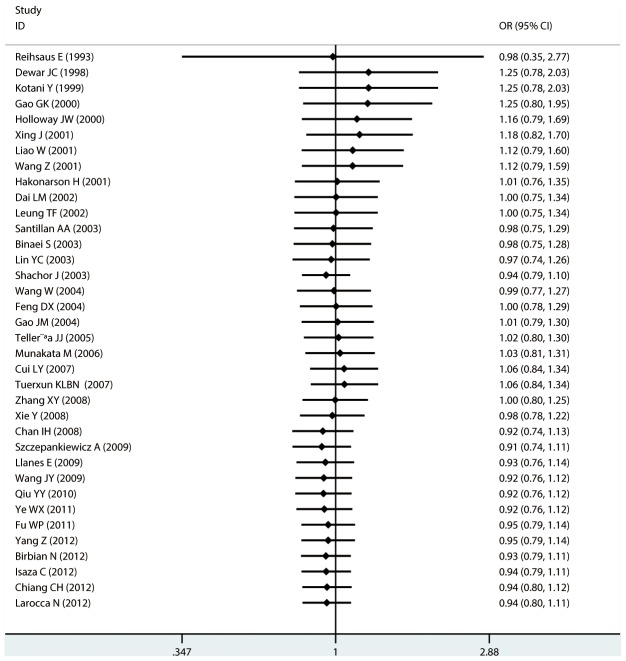
Forest plots of cumulative meta-analysis of Gln27Glu (rs1042714) in association with asthma by published year under homozygote genotype comparison.

**Figure 17:**
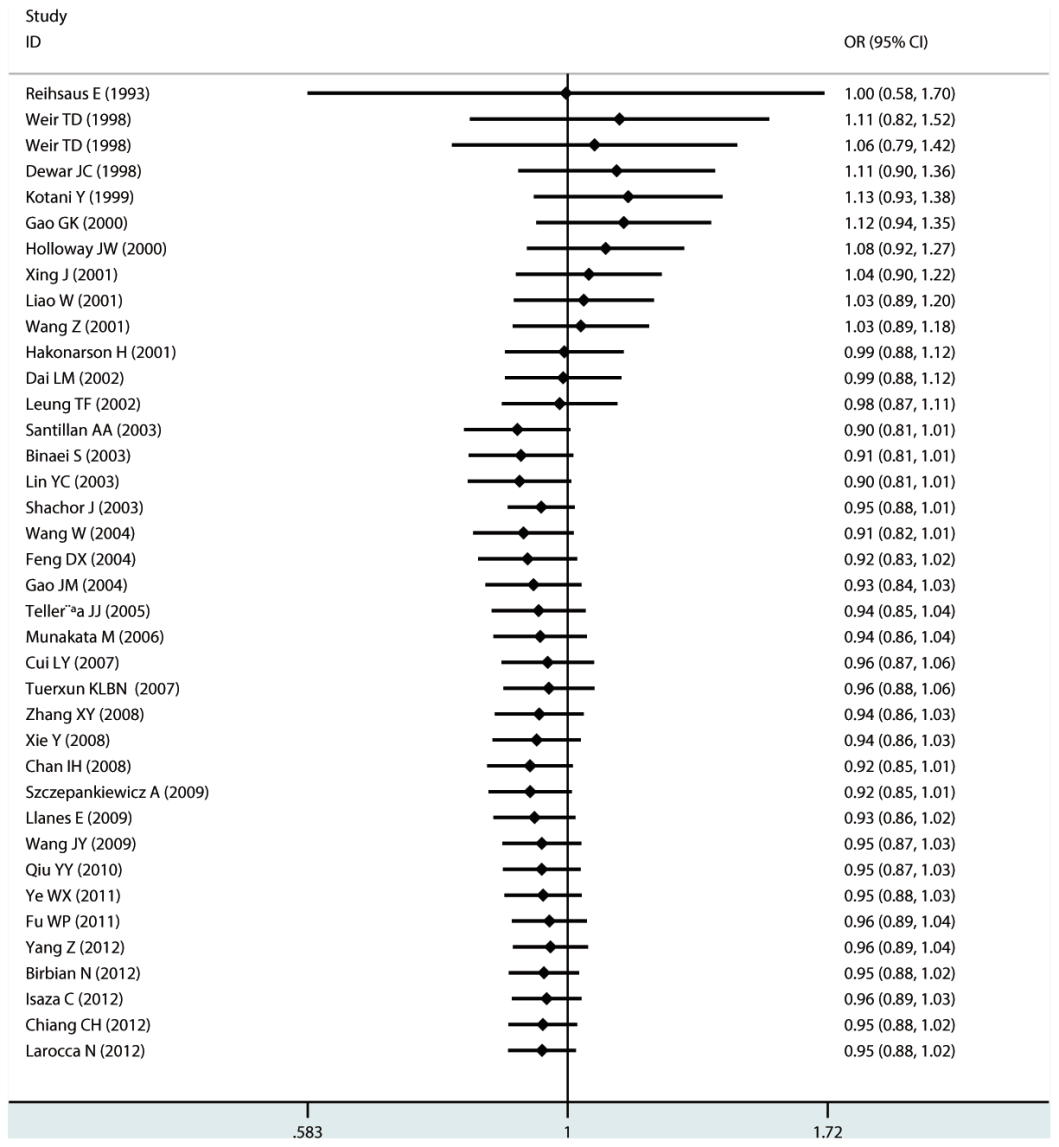
Forest plots of cumulative meta-analysis of Gln27Glu (rs1042714)in association with asthma by published year under allele comparison.

Cumulative analysis of the association between Arg16Gly and Gln27Glu polymorphisms and the risk of asthma was performed after sorting by publication date. As shown in Figures 10 to 13, for Arg16Gly, there was a stable trend in the estimated risk effect in the dominant model comparison from 2009 to 2012 and in the allelic comparison from 1993 to 2012. As shown in Figures 14 to 17, for Gln27Glu, there was a trend toward no significant association over time in all genetic model comparisons.

### Sensitivity analysis

Sensitivity analysis was conducted by sequentially excluding individual studies to estimate the stability of the results. After sequentially excluding each study, statistically similar results were found.

### Publication bias

Potential publication bias was investigated using the funnel plot and was further assessed using Egger's test. Significant publication bias was detected for the Gln27Glu polymorphism in the dominant model comparison (*t* = 2.69, *P* = 0.011). No evidence of publication bias was found for the Arg16Gly, Thr164Ile, or Arg19Cys polymorphism in any of the genetic model comparisons. The results are shown in Table 7.

**Table 7:** Publication bias results of Egger's test.

SNP	Study number (n)	Dominant model comparison		Recessive model comparison		Homozygote genotype comparison		Allele comparison	
		t	*P*	t	*P*	t	*P*	t	*P*
Arg16Gly (rs1042713)	45	1.02	0.315	0.42	0.675	0.72	0.475	1.12	0.268
Gln27Glu (rs1042714)	37	2.69	0.011	0.71	0.484	1.09	0.284	1.80	0.080
Thr164Ile (rs1800888)	4	−0.37	0.746	-	-	-	-	−2.10	0.171
Arg19Cys (rs1042711)	4	−2.01	0.294	−0.78	0.579	−0.51	0.698	−0.59	0.613

## Discussion

Asthma is a well-known disease of the respiratory system that is characterized by cramps and obstruction of the small bronchus. Β2-AR binds specifically to a class of ligands that can lead to the expansion of the small airways. In the present study, the relationship between all related *ADRB2* gene polymorphisms and the overall risk of asthma was examined. The purpose of this meta-analysis was to provide more information for asthma candidate gene research, based on the hypothesis that genetic effects vary across different ethnic cohorts.

Four ADRB2 polymorphisms that had been investigated in at least three case-control studies were included in the study. The results indicated that Arg16Gly, Gln27Glu, Thr164Ile, and Arg19Cys were not associated with risk of asthma in the overall population. The findings of the current study are consistent with those of Migita [14] and Contopoulos-Ioannidis [6]. Migita and his colleagues performed a meta-analysis by a random-effects model that showed a non-significant odds ratio for the Arg16Gly and the Gln27Glu polymorphism. Contopoulos-Ioannidis found that polymorphisms of ADRB2 are not major risk factors for the development of asthma. Cumulative analysis further confirmed that there was no significant association between the Arg16Gly polymorphism or the Gln27Glu polymorphism and the risk of asthma, showing that the variants had no effect with the accumulation of more data over time.

In the analysis stratified by case age, a protective effect for the Gln27Glu polymorphism was observed in adults in the dominant model comparison and in children in the recessive model comparison and the homozygote genotype comparison. This finding corroborates the ideas of Ammarin Thakkinstian, who suggested that the Gln/Glu and Glu/Glu genotypes could reduce the risk of asthma [15]. Besides, the pathogenesis of asthma in adults and children may differ, but the exact mechanism remains unknown and needs further detailed research.

In the analysis stratified by ethnicity, an increased risk of asthma was only seen with the Arg16Gly polymorphism in the South American population, and a protective effect was only found with the Gln27Glu polymorphism in the North American population and only in the dominant model comparison. The discrepancies in linkage disequilibrium (LD) structure in Chinese and Europeans may explain these differences: the minor allele of the ADRB2 Arg16Gly (A46G, rs1042713) in the population of northern and western European ancestry (CEU) was A with a frequency of 0.358, whereas it was G with a frequency of 0.439 among the Han Chinese in Beijing (HCB). The minor allele of the ADRB2 Gln27Glu (C79G, rs1042714) was 0.467, whereas it was 0.122 in HCB. Another reason for these differences is that sample size was small for the South American and North American populations, and therefore the current boundary result may have been unable to demonstrate that the Arg16Gly and Gln27Glu polymorphisms are associated with the risk of asthma in these populations. More studies with a larger sample size are needed. In the Chinese population, the results of the current meta-analysis showed that there was no significant association with the risk of asthma with either the Arg16Gly polymorphism or the Gln27Glu polymorphism in any of the genetic model comparisons, supporting Ni Suiqin's [16] conclusion.

In the analysis stratified by HWE according to the P-value for the Arg16Gly and Gln27Glu polymorphisms, a significant association was found in the recessive model comparison and the homozygote genotype comparison for Arg16Gly in the group with *P*<0.05, but not in the group with *P*>0.05. For Gln27Glu, a significant association was found in the dominant model comparison in the group with *P*>0.05. These results therefore need to be interpreted with caution. There are several possible explanations as to why the control group population was not in HWE. First, the population was not characterized by random mating. Second, the locus under consideration exhibited an inconstant fluctuating mutation rate. Third, there was selection for a particular phenotype. Fourth, the population was not sufficiently large or non-random. Fifth, there had been a change in the population structure during the period of study due to migration.

No significant association with the risk of asthma was found for the Thr164Ile and Arg19Cys polymorphisms. Thus, the Thr164Ile and Arg19Cys polymorphisms may not be involved in the pathogenesis of asthma. Further research is needed because, as only four case-controls were included in the study, there might not be sufficient statistical evidence to clarify the association between the Thr164Ile and Arg19Cys polymorphisms and the risk of asthma.

*ADRB2* is located on chromosome 5q31–32, encodes 413 amino acids, and is an intronless gene [5]. According to the SNPper database, there are more than 100 SNPs in the promoter region, five SNPs in the 5′UTR region and 18 SNPs in the coding region of the gene. The mutation of the two most important SNPs, Arg16Gly and Gln27Glu, which are located at nucleotide positions 46 and 79 of the coding region of the *ADRB2* gene, respectively, can cause changes in the amino acid sequence. The altered amino acid sequence can lead to down-regulation of the β2-AR and may cause the desensitization of related reactions [70]. Thr164Ile is also located in the coding region of the *ADRB2* gene; a base change from C to T can lead to a change in amino acid from threonine (Thr) to isoleucine (Ile). The missense polymorphisms of Arg16Gly, Gln27Glu, and Thr164Ile may lead to functional changes in *ADRB2*. Most of the studies relating to *ADRB2* and asthma risk have focused on coding region polymorphisms. In recent years, studies on *ADRB2* have not been confined to coding region polymorphisms alone, as more and more studies have begun to pay attention to promoter region polymorphisms. Arg19Cys is located in the 5′ leader region that harbors an open reading frame (ORF) in the promoter region of the *ADRB2* gene; a base change from T to C leads to a change in amino acid from arginine (Arg) to cysteine app:addword:cysteine(Cys). Recent *in vivo* and *in vitro* research has demonstrated that this change can impede the translation of *ADRB2* mRNA, and thus can regulate cellular expression of the receptor [71]. Further studies are therefore required to assess whether the SNPs in *ADRB2* alter signal regulation, gene expression, or the function of its product or not.

There are certain inevitable limitations to the current meta-analysis. First, all available literature should be included in the meta-analysis, but we only included literature published in English and Chinese, thus neglecting studies published in other languages. In addition, most of the included studies just focus on Chinese and Asian, which may result in an inability to detect modest association due to lack of power because of underreporting/lower incidence of asthma in these populations. Second, most original literature only provides a generic asthma definition, and does not describe asthma phenotype(s) and environmental factors in detail, so we cannot supply this information. Third, several studies were not included because they did not provide sufficient data for statistical analysis, which may have biased the result. Fourth, publication bias was only detected for the Gln27Glu polymorphism in the dominant model comparison (*t* = 2.69, *P* = 0.011), but not in the other three genetic model comparisons. In fact, positive results or results with “expected” findings are more likely to be published. Publication bias may lead to a false positive result. We detected significant publication bias for the Gln27Glu polymorphism in the dominant model, so the results need to be interpreted with caution. Fifth, moderate heterogeneity was found in some genetic models for the Arg16Gly polymorphism. Because no information was available other than the factors we performed a stratified analysis, and thus we were unable to use meta-regression to explore other possible sources of between-group heterogeneity. Furthermore, the result of the sensitivity analysis was stable. Therefore, the heterogeneity seemed to have no effect on the results, suggesting their reliability.

In conclusion, the current meta-analysis suggests that the Arg16Gly, Gln27Glu, Thr164Ile, and Arg19Cys polymorphisms may not be involved in the risk of asthma in the overall population or the Chinese population. Well-designed, high-quality studies with a larger sample size and various ethnicities should be conducted to confirm these results.

## Supporting Information

Checklist S1 **PRISMA checklist.**
(DOC)

## References

[1] To T, Stanojevic S, Moores G, Gershon AS, Bateman ED, et al (2012) Global asthma prevalence in adults: findings from the cross-sectional world health survey. BMC Public Health 12: 204. 22429515 10.1186/1471-2458-12-204 PMC3353191

[2] Pinto LA, Stein RT, Kabesch M (2008) Impact of genetics in childhood asthma. J Pediatr (Rio J) 84: S68–75. 18690379 10.2223/JPED.1781

[3] Litonjua AA, Gong L, Duan QL, Shin J, Moore MJ, et al (2010) Very important pharmacogene summary ADRB2. Pharmacogenet Genomics 20: 64–69. 19927042 10.1097/FPC.0b013e328333dae6 PMC3098753

[4] Pignatti PF (2004) Trends in pharmacogenomics of drugs used in the treatment of asthma. Pharmacol Res 49: 343–349. 15202513 10.1016/j.phrs.2003.04.002

[5] Brodde OE, Leineweber K (2005) Beta2-adrenoceptor gene polymorphisms. Pharmacogenet Genomics 15: 267–275. 15864127 10.1097/01213011-200505000-00001

[6] Contopoulos-Ioannidis DG, Manoli EN, Ioannidis JP (2005) Meta-analysis of the association of beta2-adrenergic receptor polymorphisms with asthma phenotypes. J Allergy Clin Immunol 115: 963–972. 15867853 10.1016/j.jaci.2004.12.1119

[7] Finkelstein Y, Bournissen FG, Hutson JR, Shannon M (2009) Polymorphism of the ADRB2 gene and response to inhaled beta- agonists in children with asthma: a meta-analysis. J Asthma 46: 900–905. 19905915 10.3109/02770900903199961

[8] Gao JM, Lin YG, Qiu CC, Liu YW, Ma Y, et al (2004) Beta2-adrenergic receptor gene polymorphism in Chinese Northern asthmatics. Chin Med Sci J 19: 164–169. 15506640

[9] Chiang CH, Lin MW, Chung MY, Yang UC (2012) The association between the IL-4, ADRbeta2 and ADAM 33 gene polymorphisms and asthma in the Taiwanese population. J Chin Med Assoc 75: 635–643. 23245479 10.1016/j.jcma.2012.08.012

[10] Chan IH, Tang NL, Leung TF, Huang W, Lam YY, et al (2008) Study of gene-gene interactions for endophenotypic quantitative traits in Chinese asthmatic children. Allergy 63: 1031–1039. 18691306 10.1111/j.1398-9995.2008.01639.x

[11] Kohyama K, Abe S, Kodaira K, Yukawa T, Hozawa S, et al (2011) Arg16Gly beta2-adrenergic receptor gene polymorphism in Japanese patients with aspirin-exacerbated respiratory disease. Int Arch Allergy Immunol 156: 405–411. 21829036 10.1159/000324463

[12] Kukreti R, Bhatnagar P, B-Rao C, Gupta S, Madan B, et al (2005) Beta(2)-adrenergic receptor polymorphisms and response to salbutamol among Indian asthmatics*. Pharmacogenomics 6: 399–410. 16004558 10.1517/14622416.6.4.399

[13] Shachor J, Chana Z, Varsano S, Erlich T, Goldman E, et al (2003) Genetic polymorphisms of the beta-2 adrenergic receptor in Israelis with severe asthma compared to non-asthmatic Israelis. Isr Med Assoc J 5: 821–824. 14650112

[14] Migita O, Noguchi E, Jian Z, Shibasaki M, Migita T, et al (2004) ADRB2 polymorphisms and asthma susceptibility: transmission disequilibrium test and meta-analysis. Int Arch Allergy Immunol 134: 150–157. 15153795 10.1159/000078648

[15] Thakkinstian A, McEvoy M, Minelli C, Gibson P, Hancox B, et al (2005) Systematic review and meta-analysis of the association between {beta}2-adrenoceptor polymorphisms and asthma: a HuGE review. Am J Epidemiol 162: 201–211. 15987731 10.1093/aje/kwi184

[16] Ni SQ, Tan LW (2012) Meta-Analysis of Association between β-2 Adrenoceptor Polymorphisms and Bronchi Asthma in Chinese. Pharmacy Today 22: 159–166.

[17] Tatarskyy PF, Chumachenko NG, Kucherenko AM, Gulkovskyi RV, Arabskaya LP, et al (2011) Study of possible role of CYP1A1, GSTT1, GSTM1, GSTP1, NAT2 and ADRB2 genes polymorphisms in bronchial asthma development in children. Biopolymers and Cell 27: 66–73.

[18] Sy HY, Ko FWS, Chu HY, Chan IHS, Liu TC, et al (2010) Association between candidate genes and spirometric variables in Chinese. Paediatric Respiratory Reviews 11: S2.

[19] Chung LP, Shi J, Baltic S, Waterer G, Thompson PJ (2010) Prevalence of ADRB2 polymorphisms in caucasians with severe asthma compared with mild and non-asthmatics. Respirology 15: A42.

[20] Leung T, Ko F, Sy H, Chu H, Liu T, et al (2009) Functional ADRB2 polymorphisms are associated with asthma endophenotypes in Chinese adults but not children. Allergy: European Journal of Allergy and Clinical Immunology 64: 186.

[21] Guo XR, Gong WX, Weng YQ, Zheng Q, Li SJ, et al (2005) Association between β(2)-adrenergic receptor polymorphisms and Cough variant asthma. Chinese Journal of Primary Medicine and Pharmacy 12: 2.

[22] Ren L (2011) Association between ADAM33, LTS pathway, ADRB2 genes polymorphisms and childhood asthma [Master]. Chongqing: Chongqing Medical University.

[23] Su MW, Tung KY, Liang PH, Tsai CH, Kuo NW, et al (2012) Gene-gene and gene-environmental interactions of childhood asthma: a multifactor dimension reduction approach. PLoS One 7: e30694. 22355322 10.1371/journal.pone.0030694 PMC3280263

[24] Hawkins GA, Tantisira K, Meyers DA, Ampleford EJ, Moore WC, et al (2006) Sequence, haplotype, and association analysis of ADRbeta2 in a multiethnic asthma case-control study. Am J Respir Crit Care Med 174: 1101–1109. 16931635 10.1164/rccm.200509-1405OC PMC2648111

[25] Fu J, Chen H, Hu L, Zhang H, Ma Y, et al (2002) Association between the genetic polymorphisms of beta2-adrenergic receptor gene and the asthma susceptibility and clinical phenotypes in a Chinese population. Zhonghua Yi Xue Yi Chuan Xue Za Zhi 19: 41–45. 11836685

[26] Liggett SB (1997) Polymorphisms of the beta2-adrenergic receptor and asthma. Am J Respir Crit Care Med 156: S156–162. 9351598 10.1164/ajrccm.156.4.12tac-15

[27] Huo J (2011) A study on the verification of the predisposing gene model for predicting childhood asthma in Han nationality[Master]. Shanghai: Shanghai Jiaotong University.

[28] Cichy M, Adamek-Guzik T, Uracz D, Uracz W, Czerniawska-Mysik G, et al (2005) [The functional relevance of Arg16Gly and Gln27Glu-beta2-adrenoreceptor polymorphism in patients with asthma and allergic rhinitis]. Folia Med Cracov 46: 33–51. 17037286

[29] Cui LY, Liu XH, Gao LX, Fan DS (2007) Study on the Association between β2-adrenergic Receptor Genetic polymorphisms and asthma in the population of Inner Mongolia. Chinese journal of Clinical Medicin 14: 477–481.

[30] Ye WX, Feng DX, Zhang XY, Yu H, Xu M (2011) Study on the Relationship between β2-adrenergic Receptor Genetic polymorphisms and asthma a in Miao Nationality. Journal of Medical Research 40: 83–85.

[31] Zhang XY, Zhao WL, Gui Q, He NH (2008) Relationship between genetic polymorphisms of β2-adrenergic receptor and childhood asthma. Journal of Clinical Pediatrics 26 : 399–402+408.

[32] Wang W, Wufuer HMTL, Shabiti YLHMJ, Xiang YB, Abula ABLKM (2004) Association between the genetic polymorphisms of β2-adrenergic receptor gene and the asthma susceptibility and clinical phenotypes in Uygur population. Journal of Cardiovascular and Pulmonary Diseases 23: 147–152.

[33] Yang Z, Zhang H, Wang W, Yin Y, Zhang L, et al (2012) Effect of β2-adrenergic receptor polymorphisms on childhood asthma and therapeutic efficacy of long acting β2-agonist. Journal of Clinical Pediatrics 30: 739–743.

[34] Feng DX, Ye WX, Zhang XY, Yu H, Diao XY, et al (2004) Study on β2-adrenergic Receptor Genetic Polymorphisms and Asthma. Journal of Modern Clinical Medical Bioengineering 10: 5–7.

[35] He XQ, Li FX, Tan JY, Yang XX (2012) Association of single nucleotide polymorphisms of ADRB2 Arg16Gly with asthma in southern Chinese population. Immunological Journal 28 : 687–690+702.

[36] Xie Y, Yang ZZ, Chai BC (2008) Relationship of Genetic Polymorphisms of β2 - Adrenergic Receptor and Asthma in Children in Shanghai Area. Journal of Applied Clinical Pediatrics 23 : 272–273+303.

[37] Xing J, Wang C, Liu JZ, Yan M, Huang KW, et al (2001) Association of receptor gene polymorphisms with asthma in Northern Chinese Han Population. Chinese Journal of Internal Medicine 40: 3.12953684

[38] Liu L, Fang LZ, Dai LM (2009) Combination Effect of Gene Polymorphisms in 16 Position of β2-adenergic Receptor and Cigarette Smoking on Asthma in Chinese Han Individuals. Medical Recapitulate 15: 4.

[39] Dai LM, Wang ZL, Zhang YP, Li W, Zhao ZH, et al (2002) Association of beta2 receptor gene polymorphisms with lung function in asthma patients. Chinese Journal of Tuberculosis and Respiratory Diseases 25: 2.

[40] Dai XH, Zhou JP (2008) Association of IL-13 and beta2 receptor gene polymorphisms with asthma. Shandong Medical Journal 48: 3.

[41] Liao W, Li WM, Zhao CM, Guang LX, Yin XJ, et al (2001) Preliminary Study on the relationship between β2-adrenergic receptors genetic polymor- phisms and asthma in children of Han nationality of Chongqing. Journal of Third Military Medical University 23: 968–971.

[42] Tuerxun KLBN, Shabiti YLHM, Wang W, Wufuer HMTL (2007) Study on the β2AR polymorphism in asthmatic abnormal black savda patients. Journal of Xinjiang Medical University30: 945–948.

[43] Zheng BQ, Wang GL, Yang S, Lu YQ, Liu RJ, et al (2012) [Study of genetic susceptibility in 198 children with asthma]. Zhongguo Dang Dai Er Ke Za Zhi 14: 811–814. 23146724

[44] Birbian N, Singh J, Jindal SK, Singla N (2012) Association of beta(2)-adrenergic receptor polymorphisms with asthma in a North Indian population. Lung 190: 497–504. 22821646 10.1007/s00408-012-9407-7

[45] Isaza C, Sepulveda-Arias JC, Agudelo BI, Arciniegas W, Henao J, et al (2012) beta(2) -adrenoreceptor polymorphisms in asthmatic and non-asthmatic schoolchildren from Colombia and their relationship to treatment response. Pediatr Pulmonol 47: 848–855. 22328447 10.1002/ppul.22521

[46] Fu WP, Zhao ZH, Zhong L, Sun C, Fang LZ, et al (2011) Relationship between polymorphisms in the 5′ leader cistron, positions 16 and 27 of the adrenergic beta2 receptor gene and asthma in a Han population from southwest China. Respirology 16: 1221–1227. 21801278 10.1111/j.1440-1843.2011.02028.x

[47] Qiu YY, Zhang XL, Qin Y, Yin KS, Zhang DP (2010) Beta(2)-adrenergic receptor haplotype/polymorphisms and asthma susceptibility and clinical phenotype in a Chinese Han population. Allergy Asthma Proc 31: 91–97. 20929600 10.2500/aap.2010.31.3371

[48] Szczepankiewicz A, Breborowicz A, Sobkowiak P, Kramer L, Popiel A (2009) Role of ADRB2 gene polymorphism in asthma and response to beta(2)-agonists in Polish children. J Appl Genet 50: 275–281. 19638684 10.1007/BF03195683

[49] Llanes E, Quiralte J, Lopez E, Sastre B, Chacartegui M, et al (2009) Analysis of polymorphisms in olive pollen allergy: IL13, IL4RA, IL5 and ADRB2 genes. Int Arch Allergy Immunol 148: 228–238. 18849614 10.1159/000161583

[50] Munakata M, Harada Y, Ishida T, Saito J, Nagabukuro A, et al (2006) Molecular-based haplotype analysis of the beta 2-adrenergic receptor gene (ADRB2) in Japanese asthmatic and non-asthmatic subjects. Allergol Int 55: 191–198. 17075257 10.2332/allergolint.55.191

[51] Tsai HJ, Shaikh N, Kho JY, Battle N, Naqvi M, et al (2006) Beta 2-adrenergic receptor polymorphisms: pharmacogenetic response to bronchodilator among African American asthmatics. Hum Genet 119: 547–557. 16596417 10.1007/s00439-006-0169-2

[52] Telleria JJ, Blanco-Quiros A, Muntion S, Antonio Garrote J, Arranz E, et al (2006) Tachyphylaxis to beta2-agonists in Spanish asthmatic patients could be modulated by beta2-adrenoceptor gene polymorphisms. Respir Med 100: 1072–1078. 16263254 10.1016/j.rmed.2005.09.028

[53] Bhatnagar P, Gupta S, Guleria R, Kukreti R (2005) beta2-Adrenergic receptor polymorphisms and asthma in the North Indian population. Pharmacogenomics 6: 713–719. 16207148 10.2217/14622416.6.7.713

[54] Santillan AA, Camargo CA Jr, Ramirez-Rivera A, Delgado-Enciso I, Rojas-Martinez A, et al (2003) Association between beta2-adrenoceptor polymorphisms and asthma diagnosis among Mexican adults. J Allergy Clin Immunol 112: 1095–1100. 14657864 10.1016/j.jaci.2003.09.029

[55] Gao G, Wang S, Zhang J (2000) [Study on beta 2 adrenergic receptor genetic polymorphisms in asthmatics in the people of the Han nationality of northern China]. Zhonghua Jie He He Hu Xi Za Zhi 23: 93–97. 11778498

[56] Wang Z, Chen C, Niu T, Wu D, Yang J, et al (2001) Association of asthma with beta(2)-adrenergic receptor gene polymorphism and cigarette smoking. AmJ Respir Crit Care Med 163: 1404–1409. 11371409 10.1164/ajrccm.163.6.2001101

[57] Holloway JW, Dunbar PR, Riley GA, Sawyer GM, Fitzharris PF, et al (2000) Association of beta2-adrenergic receptor polymorphisms with severe asthma. Clin Exp Allergy 30: 1097–1103. 10931116 10.1046/j.1365-2222.2000.00929.x

[58] Reihsaus E, Innis M, MacIntyre N, Liggett SB (1993) Mutations in the gene encoding for the beta 2-adrenergic receptor in normal and asthmatic subjects. Am J Respir Cell Mol Biol 8: 334–339. 8383511 10.1165/ajrcmb/8.3.334

[59] Kocabas NA, Kaymak C, Aydin N, Oztuna D, Karakaya AE (2007) Investigation of the beta 2-adrenoceptor (ADRB2) 16 and glutathione S-transferase P1 (GSTP1) gene polymorphisms in Turkish asthma patients. Toxicology Letters 172: S164–S165.

[60] Larocca N, Moreno D, Garmendia JV, Velasquez O, Martin-Rojo J, et al (2012) Beta 2 adrenergic receptor polymorphisms, at codons 16 and 27, and bronchodilator responses in adult Venezuelan asthmatic patients. Biomed Pap Med Fac Univ Palacky Olomouc Czech Repub 157: 374–378. 23128817 10.5507/bp.2012.084

[61] Wang JY, Liou YH, Wu YJ, Hsiao YH, Wu LS (2009) An association study of 13 SNPs from seven candidate genes with pediatric asthma and a preliminary study for genetic testing by multiple variants in Taiwanese population. J Clin Immunol 29: 205–209. 18931892 10.1007/s10875-008-9256-6

[62] Binaei S, Christensen M, Murphy C, Zhang Q, Quasney M (2003) Beta2-adrenergic receptor polymorphisms in children with status asthmaticus. Chest 123: 375S. 12628991

[63] Kotani Y, Nishimura Y, Maeda H, Yokoyama M (1999) Beta2-adrenergic receptor polymorphisms affect airway responsiveness to salbutamol in asthmatics. J Asthma 36: 583–590. 10524541 10.3109/02770909909087295

[64] Weir TD, Mallek N, Sandford AJ, Bai TR, Awadh N, et al (1998) beta2-Adrenergic receptor haplotypes in mild, moderate and fatal/near fatal asthma. Am J Respir Crit Care Med 158: 787–791. 9731005 10.1164/ajrccm.158.3.9801035

[65] Dewar JC, Wheatley AP, Venn A, Morrison JF, Britton J, et al (1998) Beta2-adrenoceptor polymorphisms are in linkage disequilibrium, but are not associated with asthma in an adult population. Clin Exp Allergy 28: 442–448. 9641570 10.1046/j.1365-2222.1998.00245.x

[66] Hakonarson H, Bjornsdottir US, Ostermann E, Arnason T, Adalsteinsdottir AE, et al (2001) Allelic frequencies and patterns of single-nucleotide polymorphisms in candidate genes for asthma and atopy in Iceland. Am J Respir Crit Care Med 164: 2036–2044. 11739132 10.1164/ajrccm.164.11.2101086

[67] Leung TF, Tang NL, Chan IH, Li AM, Ha G, et al (2002) Distribution in allele frequencies of predisposition-to-atopy genotypes in Chinese children. Pediatr Pulmonol 34: 419–424. 12422339 10.1002/ppul.10210

[68] Lin YC, Lu CC, Shen CY, Lei HY, Guo YL, et al (2003) Roles of genotypes of beta2-adrenergic receptor in the relationship between eosinophil counts and lung function in Taiwanese adolescents. J Asthma 40: 265–272. 12807170 10.1081/jas-120018323

[69] Lv J, Liu Q, Hua L, Dong X, Bao Y (2009) Association of five single nucleotide polymorphism loci with asthma in children of Chinese Han nationality. J Asthma 46: 582–585. 19657898 10.1080/02770900902915847

[70] Green SA, Turki J, Bejarano P, Hall IP, Liggett SB (1995) Influence of beta 2-adrenergic receptor genotypes on signal transduction in human airway smooth muscle cells. Am J Respir Cell Mol Biol 13: 25–33. 7598936 10.1165/ajrcmb.13.1.7598936

[71] Parola AL, Kobilka BK (1994) The peptide product of a 5′ leader cistron in the beta 2 adrenergic receptor mRNA inhibits receptor synthesis. J Biol Chem 269: 4497–4505. 8308019

